# Young adults in eastern Germany know dandelion and sparrows but few farmland species

**DOI:** 10.1186/s13002-026-00908-2

**Published:** 2026-05-14

**Authors:** Naaf Tobias, Kernecker Maria

**Affiliations:** Leibniz Centre for Agricultural Landscape Research, Eberswalder Straße 84, 15374 Müncheberg, Germany

**Keywords:** Agricultural landscape, Arable plants, Biodiversity conservation, Local knowledge, Cultural domain, Farmland birds, Freelisting, Grassland plants, Nature disconnection

## Abstract

**Background:**

The global biodiversity crisis comprises the ongoing loss of species simultaneous to the decrease in local knowledge of native plant and animal species. Such local knowledge is fundamental to societal engagement in conservation efforts. It is, however, unclear how familiar people are with the biodiversity in their surrounding landscape.

**Methods:**

We used a cultural domain analysis to assess the public’s local knowledge of plant and bird species in northwest Saxony, an agricultural region in eastern Germany. We interviewed 463 adults and asked them to freelist all wild plants and birds they know to occur in their surrounding agricultural landscape. We hypothesized that (i) people know mostly common generalist species, but few species characteristic for their surrounding agricultural land; and (ii) younger people know fewer species than older people.

**Results:**

We found that 15 plant and 21 bird taxa were part of the people’s cultural domain of local farmland species. On average, people named only two plant and three bird taxa out of the 62 plant and 25 bird farmland indicator species that occur in the region. Thus, most of the species characteristic for agricultural habitats were unknown. Species knowledge generally increased with age at least up to 46–55 years. Characteristic farmland plant species such as Cornflower, Chamomile, or Yarrow, and characteristic farmland bird species such as Starling or Skylark were significantly more salient among older participants (> 45 years old), whereas generalist plants, such as Dandelion, Common daisy, or Stinging nettle, and high-order bird taxa, such as crows, pigeons and birds of prey, were more salient among younger participants (≤ 45 years old).

**Conclusions:**

Our study revealed that people’s local knowledge of plant and bird species comprises a few plant and a few more bird species that are characteristic for their agricultural landscape. Nevertheless, most of the common indicator species for agricultural habitats in our study region are not part of the people’s cultural domain. Possibilities to enhance local knowledge necessary for conserving biodiversity include restoring the cultural relevance of wild plants, as foods or medicine, and teaching the relevance of biodiversity for ecosystem functioning at school, particularly through outdoor education in rural environments.

**Supplementary Information:**

The online version contains supplementary material available at 10.1186/s13002-026-00908-2.

## Background

The global biodiversity crisis comprises two mutually amplifying processes. One is the ongoing and accelerating loss of species from this planet in response to human activities [1, 2]. The other process is the loss of local environmental and species knowledge due to progressing estrangement of people from nature, particularly in high-income countries [3–5]. A life in urban environments, and the omnipresence of technical devices in all parts of life results in the loss of human-nature interactions, referred to as the “extinction of experience” [6]. While the disappearance of organisms from our environments hampers the chance to encounter and interact with these organisms, i.e. to experience nature [7, 8], the increasing disconnection from nature leads to a fading local knowledge of biodiversity and a lack in the will and ability to conserve it [9–11]. In the end, people will only care for what they know [4, 12], and how they know different species has, until recently, been associated with everyday practices rooted in reciprocal, place-based relationships [13]. Therefore, the challenge is to maintain local knowledge about species and their life history as a part of culture to ensure the long-term conservation of biodiversity and life systems.

Conservationists are particularly concerned about the loss of local species knowledge amongst school-aged children in high-income and highly urbanized countries. Studies in these countries have shown that school children can list on average less than ten plant and animal species [16, 19, 30, 71]; often use unspecific terms to name plants and animals, such as “grass”, “trees”, “mouse”, “duck”, or “fish”, rather than species names [14–17]; know exotic or even fictitious species better than native species [12, 18–21]; and can identify less than half of the most common native animal species [10, 12, 22–25]. The ability of adults to identify common native plant and animal species was also assessed as poor [9, 23, 24, 26–29]. Plants are particularly unknown when compared with mammals or birds [14, 16, 24, 27, 30, 31]. This phenomenon is often referred to as “plant awareness disparity” [32]. It describes a cognitive and perceptual bias in which people fail to notice, appreciate, or understand the importance of plants in their environment [32], with implications for conservation, since plants form the basis for ecosystem functioning [33].

Whether local species knowledge is in fact being lost over the generations, as signature of a progressing disconnection from nature, is unclear [5]. Children appear generally to know less species than adults [11, 23, 24, 34–37] and younger adults appear to know less species than older adults [11, 23, 28, 34, 36, 37]. However, there is so far very limited evidence for a decline over age cohorts [22]. A decline in species knowledge over time is problematic as it may lead to a shifting baseline syndrome, in that younger generations with a poor knowledge of plants and animals and their life history will be less likely to recognize changes in natural conditions [4, 8]. Knowing species as the basic unit of biodiversity is generally regarded as a fundamental prerequisite for understanding species’ roles in ecological and cultural relationships, and for protecting biodiversity [15, 19, 22, 29, 38]. Furthermore, a lack of familiarity with the natural environment may undermine people’s support for pro-biodiversity policies and management actions [39]. Recent research has shown that people with a higher species knowledge have more positive attitudes towards nature and are more motivated to engage in biodiversity conservation [10, 11, 28]. Therefore, maintaining local species knowledge is elemental for maintaining our natural environments.

Previous studies generally indicate a lack of local species knowledge, particularly among younger generations in high-income countries, but they provide an incomplete picture of the extent to which such knowledge is still present or already lost. Assessments of people’s local species knowledge have largely been based on identification tests with a selected number of photos from the most common and widely spread plant and animal species (e.g. [12, 25–27]), . Furthermore, these studies were often conducted in urban environments [9, 11, 22, 27, 36]. It has been suggested that people in rural areas might be less disconnected from their surrounding nature since they have more opportunities to interact with nature [6]. While some studies found no clear differences between urban and rural communities [22, 30, 40], others observed higher species identification skills among rural participants [24, 26, 28]. However, these comparisons were based on very few common habitat generalists, although habitat specialists are those of biocultural conservation value [41], as they have evolved in response to traditional land use over millennia [42]. Getting a sense of whether people are familiar with habitat specialists would provide important information for their conservation.

To which degree people have local knowledge about habitat specialists is unclear, as is the best method for assessing this. Species identification tasks with photos of selected plant or animal species yield a quantitative estimate of the people’s identification skills, as does pointing out specific species in walking interview approaches. However, these exercises would not necessarily reveal which other species that are neither in photographs nor appear during a walk people know or do not know. Another approach to assessing people’s local knowledge is the freelisting technique which, although common in ethnobiological research [43], has been comparably rarely applied in studies assessing local species knowledge in high-income countries [16, 19, 30, 44]. This technique cannot generate people’s specific species identification skills, however it identifies which species do or do not belong to the people’s cultural domain [45] and associated local knowledge. The cultural domain is a list of things that, according to people in a certain group, somehow go together [46]. For instance, investigating which items people think belong to the domain “native wild plant species” may provide important information for conservationists [19]. Moreover, it may be important for society to reflect on the degree to which its culture has an active relationship to its landscape and the biodiversity therein.

(Agri)cultural landscapes are the results of co-evolution between humans and the landscape; human demographics, sociopolitical change and technological development have elicited responses at genetic, taxonomic and functional levels of biodiversity [47]. Agricultural landscapes are therefore biocultural places, where human culture and biodiversity coexist. In Europe however, agricultural landscapes have seen a dramatic decline in biodiversity over the last century, because of habitat loss and land-use intensification [48–50]. For instance, arable plants in Germany declined heavily in cover and diversity between the 1950s and 2009 [51]. The abundance of grassland arthropods declined by 78% between 2008 and 2017 [52]. As a result, farmland birds also declined between 1990 and 2018 with insectivorous species being most affected [53]. To assess whether those species characteristic for agricultural landscapes will continue to decline largely unnoticed by the public, or whether there is a chance to reach a societal consensus on the need to conserve the remaining farmland biodiversity, we need to understand what the local knowledge is of people living in agricultural regions in regard to their surrounding landscape. Previous research on farmers’ plant knowledge indicates that they are more familiar with species characteristic of typical agricultural habitats, either because they still have a cultural relationship with these species [35] or they simply pay more attention to those species unique to their local environment [54]. Hardly any study on people’s species knowledge has focused on specific habitats (but see [44]). As such, it is critical to gain insight into cultural knowledge on specialists in agricultural landscapes, as these species are emblematic of human-landscape co-evolution.

Here, we focussed on a typical agricultural landscape in eastern Germany and aimed to assess people’s local knowledge of their surrounding biodiversity. We were particularly interested in people’s knowledge of species characteristic for agricultural land, i.e. grasslands and arable fields. This interest emerged from farmers’ explicit wish that the wider public be more connected to and engaged in agricultural landscapes and thereby better equipped to support farmers’ efforts to conserve biodiversity [55]. Based on previous research, we expected that (i) people know mostly common generalist species, while they are unfamiliar with the majority of species characteristic for their surrounding agricultural land; and (ii) younger people know fewer species, in particular fewer species characteristic for their agricultural landscape, than older people.

## Methods

### Design and setting

We conducted our survey in two small towns in northwest Saxony, Germany, namely Delitzsch (c. 26,000 inhabitants) and Eilenburg (c. 16,500 inhabitants). The two towns are surrounded by a typical Central European agricultural landscape that is dominated by arable fields (57%) and to a lesser extent by grassland (14%) [56]. We approached potential participants on highly frequented streets or town squares. The people interviewed live either directly in one of the towns or in different small villages throughout the region from where they visited the towns for their shopping, health, and administrative needs. Our sample is thus not purely rural or urban but provides insights into a context in which most German people live – largely in between rural or urban settings [57]. In total, we interviewed 463 participants, 234 in Delitzsch and 229 in Eilenburg. All participants gave consent and were aware that they could stop at any point during the interview. Interviews lasted ten to 20 min depending on the participant.

To find out how familiar people are with the biodiversity in their surrounding agricultural landscape, we used the freelisting technique ([58]: pp. 147–151). This means we asked the participants to list all plant and bird species they could think of, and we noted the listed items down in the order they came to mind. Specifically, we asked: “think about the meadows, pastures, and field margins in your landscape. Which wildflowers or herbs can you find there?” and “think about the meadows, pastures, and arable fields in your landscape. Which bird species can you observe there?” We focused on herbaceous plants and birds because these groups comprise many species and can be most easily observed when out in the field. Therefore, we expected people to be most familiar with these groups. To make sure that the obtained freelists were as complete as possible, we followed a two-step protocol in each freelisting task [59]. First, when an interviewee indicated that they could not think of any other item, they were repeatedly asked which further items they know. Second, when they insisted that they could not remember any more items, we read the list of items slowly back to them, which often led to further items coming to mind. Moreover, if participants listed a higher-order taxon rather than a species, we asked them immediately if they would have some specific species in mind, which sometimes led to the listing of one or even several items at the species level. If participants used vernacular names unfamiliar to us, we asked them to explain which species they mean so that the listed taxa could be correctly identified in all but a few exceptional cases (see Additional file 3).

After the freelisting tasks, we asked each participant for some demographic information, i.e. their gender, age, level of education, and monthly net household income. The latter three were each measured on a five- or six-degree scale (see Additional file 1). Our aim was to obtain a sample with a uniform gender and age distribution, and we approached people intentionally, to obtain a broader representation. However, given the overrepresentation of senior citizens on the street during the day and the higher willingness of women to participate, these groups were overrepresented in our dataset (24.9% >65 years old; 60.7% women). Nevertheless, the minimum proportion of each of the six age classes among female and male participants was > 10% (see Additional file 4).

### Preparing the freelist data

To make freelist data suitable for analysis, we made item names consistent across participants. Often, people did not list species, but higher-order taxa such as a genus or a family. Other people, however, listed species from within these higher-order taxa. To make species lists comparable, we accepted such higher-order taxa as valid items, for instance “Crows” (genus *Corvus*). When another person listed one or several species from within a higher-order taxon, e.g., “Rook” (*C. frugilegus*) and/or “Hooded crow” (*C. cornix*) but not the higher-order taxon itself, we added this higher-order taxon (“Crows”) to their list and placed it just before the first item from that higher-order taxon. Thus, when it came to assessing the participants’ local species knowledge, participants knowing the higher-order taxon got one credit, while those knowing a species from within this higher-order taxon got an additional credit.

Further, we assigned items to different taxa groups. First, we considered a taxon as “valid” if it was a correct response to the question asked, i.e. a taxon that occurs regularly in open habitats (grasslands, arable fields) of the agricultural landscape in northwest Saxony, Germany, rather than an exotic species not occurring in the region or a species from other habitats, such as forest. Second, we identified indicator taxa, which can be considered characteristic for the regional agricultural landscape and are therefore of conservation value. For instance, the occurrence of grassland indicator plant species on a farmer’s grassland is a prerequisite for the reception of payments in regional agri-environmental schemes [60]. For the plant taxa, we differentiated between grassland indicator plants and arable indicator plants. Indicator plant taxa were defined as such according to regional reference lists [60–62]. Indicator bird taxa were defined according to a German reference list [63].

### Assessing people’s species knowledge

We quantified the cumulative and individual species knowledge of the 463 participants as the number of items listed by all participants together and individually, respectively. Overlapping higher-order taxa were excluded when compiling the cumulative taxa list to avoid the overestimation of knowledge. We calculated list lengths for the raw lists, valid taxa, and indicator taxa. To quantify how many plant and bird taxa belong to the people’s cultural domain we set an arbitrary frequency threshold at 5%, i.e. taxa listed by at least 5% of participants were considered part of the cultural domain. This aligns with Sutrop [45], who recommends omitting items listed by 3.75-6% of the participant number. Further, we considered those taxa listed by at least each fifth participant part of the people’s general knowledge.

To test our first hypothesis, we assessed the salience of those taxa within the cultural domain by plotting their mean rank in the freelists against their relative frequency across participants. We also calculated Smith’s S for each taxon in the domain by first dividing the inverse rank of the taxon in a freelist by the freelist length and then averaging across participants ([58]: pp 147–151).

For testing our first hypothesis, we also wanted to know which indicator species occur in the region and do or do not belong to people’s local knowledge. Therefore, we asked local experts from the regional landcare association, the Landschaftspflegeverband Nordwestsachsen e.V. (LPV), to rate how frequently each indicator species in the reference lists occurs in the region. Ratings were done on a four-degree scale: 0 – absent, 1 – rare (i.e., difficult to discover for the common citizen), 2 – occasional to regular (i.e., with some luck or effort observable for the interested citizen), 3 – common (i.e., easily observable for the common citizen during a walk or bicycle tour). Thirty-one grassland plant indicator taxa, 31 arable plant indicator taxa, and 26 farmland bird indicator taxa occur more or less frequently, i.e. with a frequency of 2 or 3, in the region (see Additional file 2).

### Effects of age

To test whether younger people know fewer plant and bird taxa than older people (second hypothesis), we modelled freelist length as a function of age and the other demographic variables using generalized additive models (GAM) with log link and negative binomial error distribution. For this purpose, we treated age, education, and income as quantitative variables. We fitted separate models for plants and birds as well as for the list length of all taxa, valid taxa, and indicator taxa. In a first step, we fitted univariate models with one quantitative demographic variable at a time to test which of the following four effect types would be most appropriate, i.e. would show the lowest AIC: (I) linear (list length ~ predictor); (II) non-linear (list length ~ s(predictor)); (III) linear with interaction (list length ~ predictor*gender); (IV) non-linear with interaction (list length ~ s(predictor, by=gender)). Then, we fitted models with all four demographic variables as predictors, each with its most appropriate effect type, and used backward selection until all remaining terms were significant at a 5% level according to a χ²-test. Models were fit with the R-functions glm.nb of the package MASS [64] and gam of the package mgcv [65].

We also wanted to know whether indicator taxa within the cultural domain are less frequently listed by younger compared to older participants. For this purpose, we calculated the relative frequency of all plant and bird indicator species within the cultural domain separately for each age class. Then, we modelled this relative frequency as a function of age using binomial generalized additive mixed models (GAMM) with taxon as random effect. Again, we treated age as a quantitative variable. Models were fit with the function gamm of the package mgcv.

Finally, to identify taxa that are more or less salient among younger and older people, we calculated the salience of all taxa within the cultural domain separately for two broad age categories: younger participants between 18 and 45 years old and older participants more than 46 years old. Then, we used two-sample randomization tests with 10,000 permutations [66] to determine whether each taxon's average salience differed between the two age categories.

## Results

### Cumulative and individual species knowledge

**Fig. 1:**
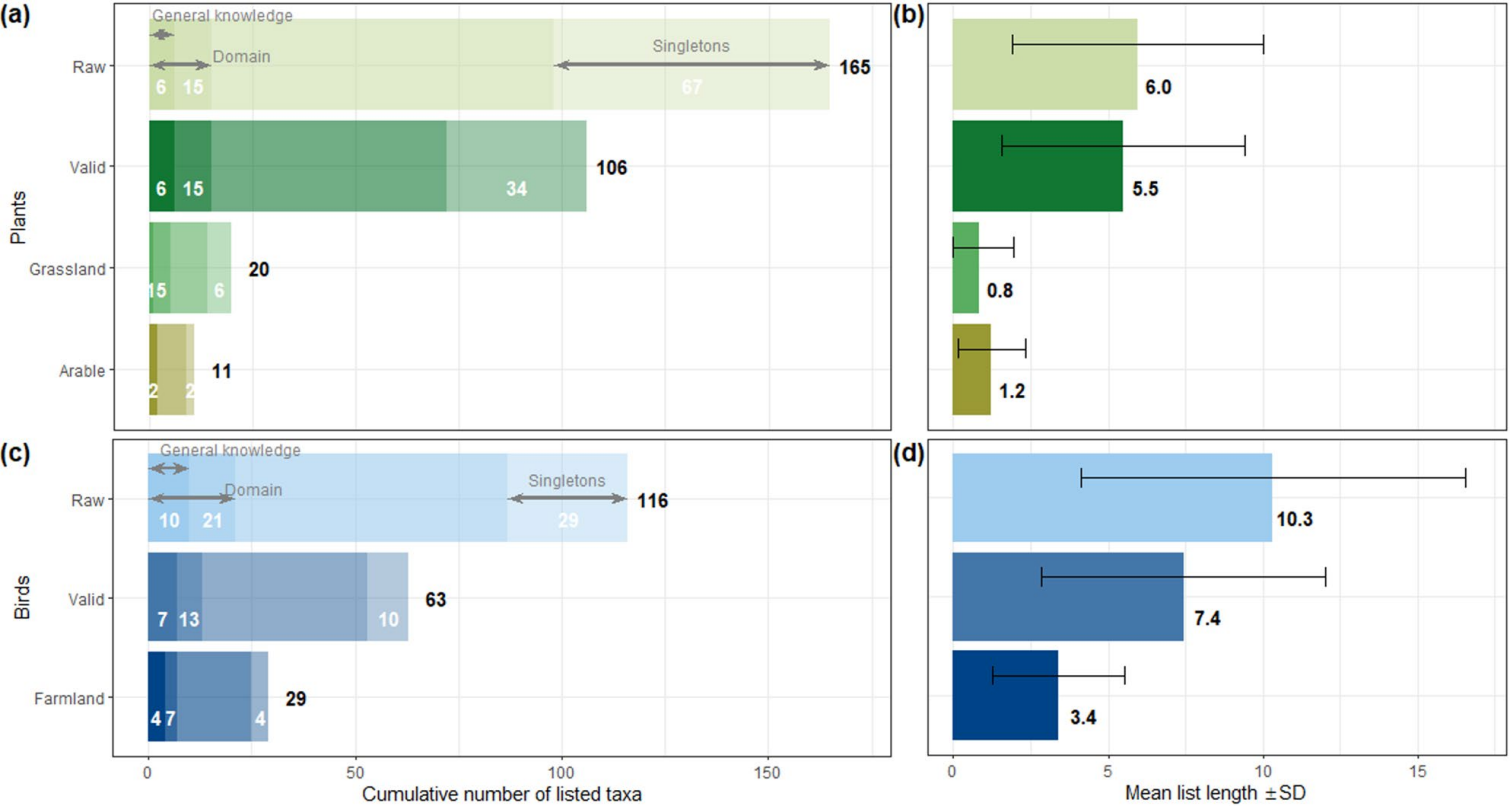
Cumulative species knowledge of all 463 participants (**a**, **c**) and mean individual freelist length for the different species groups (**b**, **d**). Error bars show the standard deviation

All participants together listed a total of 165 plant and 116 bird taxa (any higher-order taxa excluded; Additional file 3). However, 41% and 25% of these, respectively, were listed by a single participant only (Fig. 1a, c). Only 15 plant taxa and 21 bird taxa can be considered part of the cultural domain (i.e., were listed by at least 5% of the people), while 6 plant and 10 bird taxa can be considered part of the people’s general local knowledge (i.e., were listed by at least each fifth participant). All 15 plant taxa in the cultural domain and 13 out of the 21 bird taxa in the cultural domain were “valid”, i.e. represented correct responses to our question and thus occur as wild-living populations in the open agricultural landscape of the region rather than in forests or gardens. Among these valid taxa, there were five grassland plant indicator taxa, two arable plant indicator taxa, and seven farmland bird indicator taxa.

Individual list length varied considerably from 0 to 23 plant taxa and from 0 to 35 bird taxa. On average, participants listed 6.0 plant taxa and 10.3 bird taxa, of which 5.5 and 7.4 were valid, respectively (Fig. 1b, d). The average participant listed 2.1 plant indicator taxa (0.8 grassland and 1.2 arable field taxa) and 3.4 bird indicator taxa. Freelist lengths for plant and bird species were correlated (*r* = 0.67, *p* < 0.001) but also showed considerable independent variation (see Additional file 5) indicating that people who know many plants do not necessarily know many birds and vice versa.

### Salient and unknown species

**Fig. 2:**
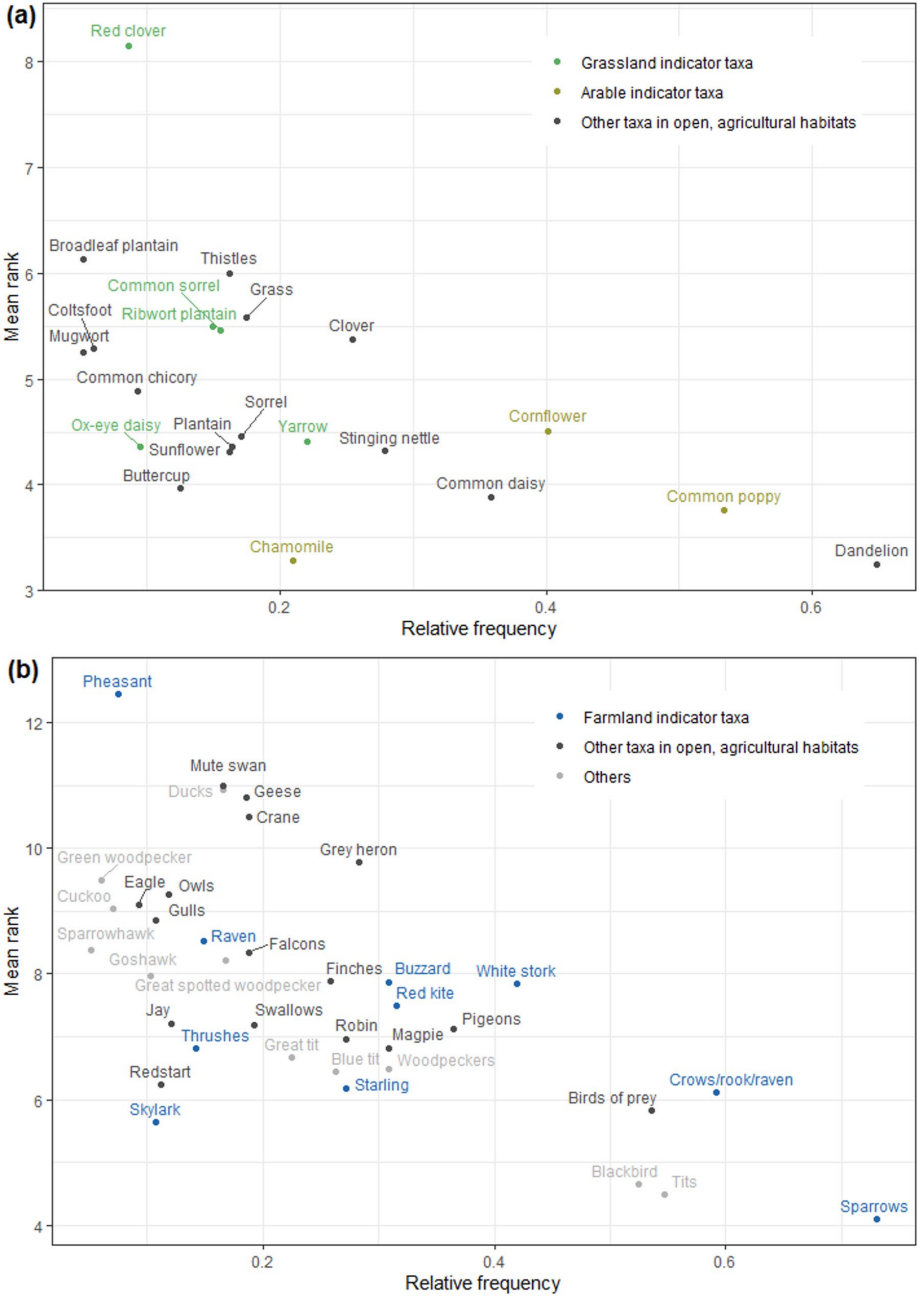
Salience of those plant (**a**) and bird (**b**) taxa listed by more than 5% of the participants

If we take overlapping higher-order taxa into account, 22 plant and 37 bird taxa belong to the cultural domain (Fig. 2a, b). Eight plant taxa can be considered part of the general knowledge of the participants, i.e. were listed by at least each fifth participant (in order of decreasing salience): Dandelion (*Taraxacum* sect. Ruderalia), Common poppy (*Papaver rhoeas*), Cornflower (*Centaurea cyanus*), Common daisy (*Bellis perennis*), Stinging nettle (*Urtica dioica*), Clover (*Trifolium* spp.), Yarrow (*Achillea millefolium*), Chamomile (*Matricaria* spp./*Tripleurospermum* spp.). Seventeen bird taxa can be considered part of the general knowledge: sparrows (*Passer* spp.), crows/rook/raven (*Corvus* spp.), tits (Paridae), birds of prey (Accipitriformes), Blackbird (*Turdus merula*), White stork (*Ciconia ciconia*), pigeons (Columbidae), Red kite (*Milvus milvus*), woodpeckers (Picidae), Magpie (*Pica pica*), Buzzard (*Buteo buteo*), Grey heron (*Ardea cinerea*), Starling (*Sturnus vulgaris*), Robin (*Erithacus rubecula*), Blue tit (*Cyanistes caeruleus*), finches (Fringillidae), and Great tit (*Parus major*). The only taxon for which people used several different vernacular names was dandelion, which people called “Löwenzahn,” “Speckblume,” or “Hundeblume.”

Of the 31 grassland plant indicator taxa that occur occasionally to frequently in the region (see Additional file 2), five were within the people’s cultural domain (Fig. 2a). This means that the other 26 more or less frequent grassland indicator taxa were not at all or only exceptionally known by the local people. Examples for grassland plant indicator taxa that are common in the region but unknown by the people are *Centaurea* spp., *Crepis* spp., *Hieracium* spp., *Hypochoeris* spp., *Leontodon* spp., *Galium* spp., *Veronica chamaedrys*, *Carex* spp., or *Armeria elongata*. Of the 31 arable plant indicator taxa that occur occasionally to frequently in the region (see Additional file 2), three were within the people’s cultural domain (Fig. 2a). This means that the other 28 more or less frequent arable plant indicator taxa were not at all or only exceptionally known by the local people. Examples for arable plant indicator taxa that are common in the region but unknown by the people are *Anthemis arvensis*, *Glebionis segetum*, *Lapsana communis*, *Linaria vulgaris*, *Raphanus raphanistrum*, *Sonchus* spp., or *Viola arvensis*.

Twenty-five farmland bird species occur occasionally to frequently in the region (see Additional file 2). Seven of these are within the people’s cultural domain. Another three taxa at a higher taxonomic level (sparrows, crows, thrushes) also belong to the domain (Fig. 2b). This means that the other 19 more or less frequent farmland bird indicator species that occur in the region are not at all or only exceptionally known. Examples of bird species that are common in the region but unknown by the people are *Sylvia communis* or *Emberiza citrinella*. Some species were listed only at a higher taxonomic level: *Phoenicurus phoenicurus*, *Turdus philomelos*, and *Passer domesticus*.

### Effects of age on species knowledge

**Fig. 3:**
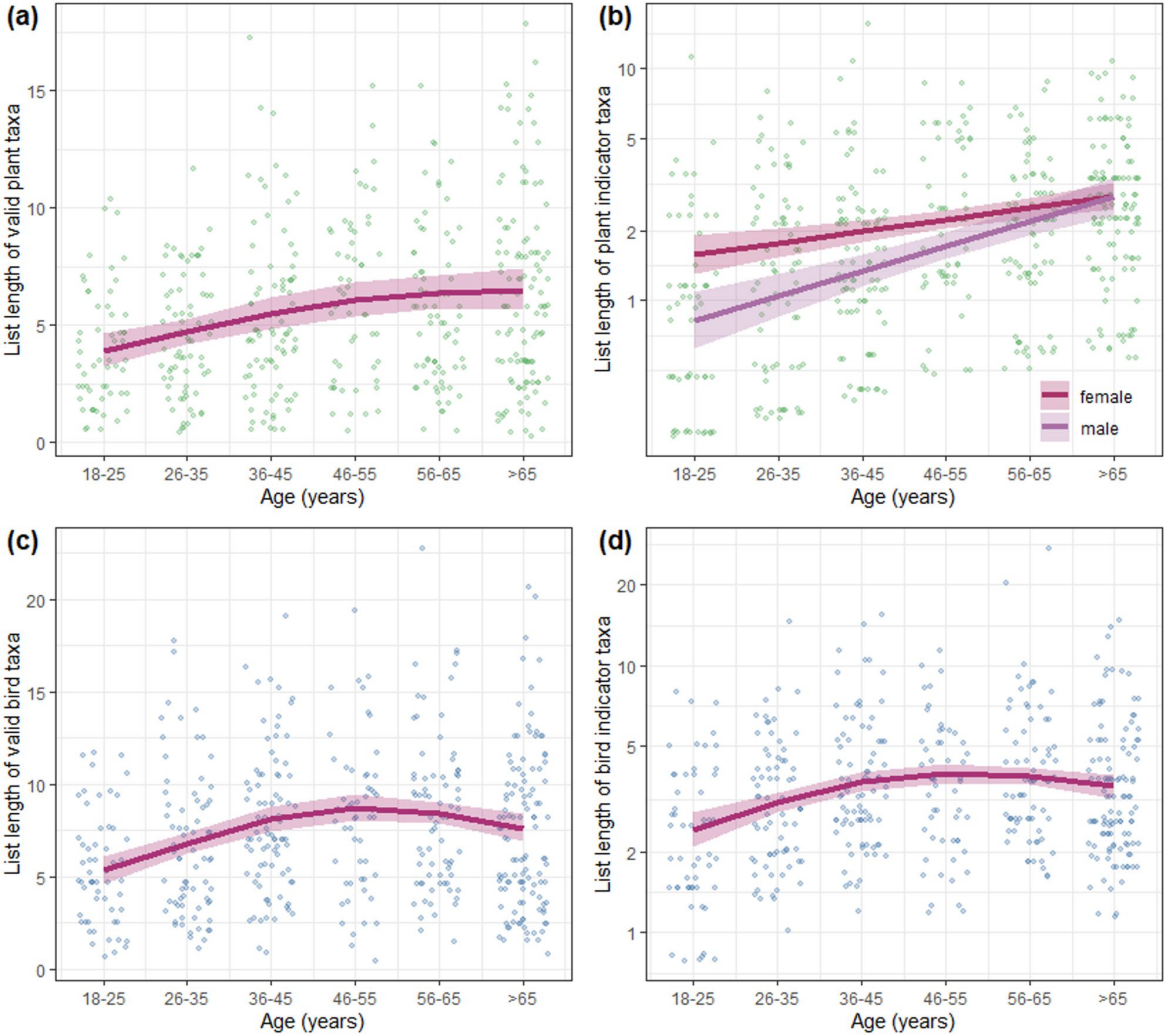
Partial effects of age on the freelist length for valid plant taxa (**a**), plant indicator taxa (**b**), valid bird taxa (**c**), and bird indicator taxa (**d**) according to generalized additive models (Table 1). Shown are partial residuals, the regression line and the 95% confidence band

The effects of age on freelist length for raw lists and lists of valid taxa were very similar (Table 1). Therefore, we focus on valid taxa and indicator taxa. Freelist length generally increased with age, particularly between the youngest (18–25 years) and the intermediate age category (46–55 years). For valid plant taxa, valid bird taxa, and bird indicator taxa, the increase levelled off or even slightly reversed beyond an age of 55 years (Fig. 3a, c, d), while for plant indicator taxa the increase was linear up to an age over 65 years (Fig. 3b). Besides age, also gender and the level of education had significant effects on freelist length. Freelist length generally increased with the level of education (see Additional file 6 a, b, d, e). Young to middle-aged female participants (≤ 55 years) listed more plant indicator taxa than did young to middle-aged male participants (Fig. 3b). In contrast, male participants generally listed more bird taxa than did female participants (see Additional file 6 c). Although we found highly significant effects of demographic variables on freelist length, freelist length showed considerable variation independent of the demography, which explained only 7.6% to 15.2% of the variation (Table 1).

**Table 1:** Final models used to explain freelist lengths of plants and birds by demographic variables. Separate models were fitted for freelists including all taxa (raw), valid taxa, or indicator taxa only. Terms surrounded by ‘s(…)’ are smooth terms for fitting non-linear effects. Given are the degrees of freedom (df), the chi-squared statistic (*χ*²), the *p*-value, the percentage of explained deviance (Dev. exp.), and the number of included participants (*n*)

Model	Term	df	χ ²	*p*	Dev. exp. (%)	*n*
LL.plants.raw ~ Education + s(Age, k = 3, fx = TRUE)					7.63	458
	s(Age)	2	20.2	< 0.001		
	Education	1	23.7	< 0.001		
LL.plants.valid ~ s(Age, k = 3, fx = TRUE) + s(Education, k = 3, fx = TRUE, by = Gender)					11.0	456
	s(Age)	2	27.6	< 0.001		
	s(Education): female	2	33.0	< 0.001		
	s(Education): male	2	2.33	0.313		
LL.plants.indicators ~ Gender * Age + Education					15.2	456
	Gender	1	13.1	< 0.001		
	Age	1	18.8	< 0.001		
	Gender: Age	1	7.19	0.007		
	Education	1	26.7	< 0.001		
LL.birds.raw ~ Gender + s(Age, k = 3, fx = TRUE) + Education					9.74	456
	Gender	1	11.9	< 0.001		
	s(Age)	2	25.6	< 0.001		
	Education	1	12.6	< 0.001		
LL.birds.valid ~ Gender + s(Age, k = 3, fx = TRUE) + Education					10.8	456
	Gender	1	14.2	< 0.001		
	s(Age)	2	27.8	< 0.001		
	Education	1	14.4	< 0.001		
LL.birds.farmland ~ Gender*Education + s(Age, k = 3, fx = TRUE)					13.3	456
	Gender	1	16.0	< 0.001		
	s(Age)	2	24.3	< 0.001		
	Education	1	27.4	< 0.001		
	Gender: Education	1	6.42	0.011		

### Familiarity with indicator species in dependence of age

**Fig. 4:**
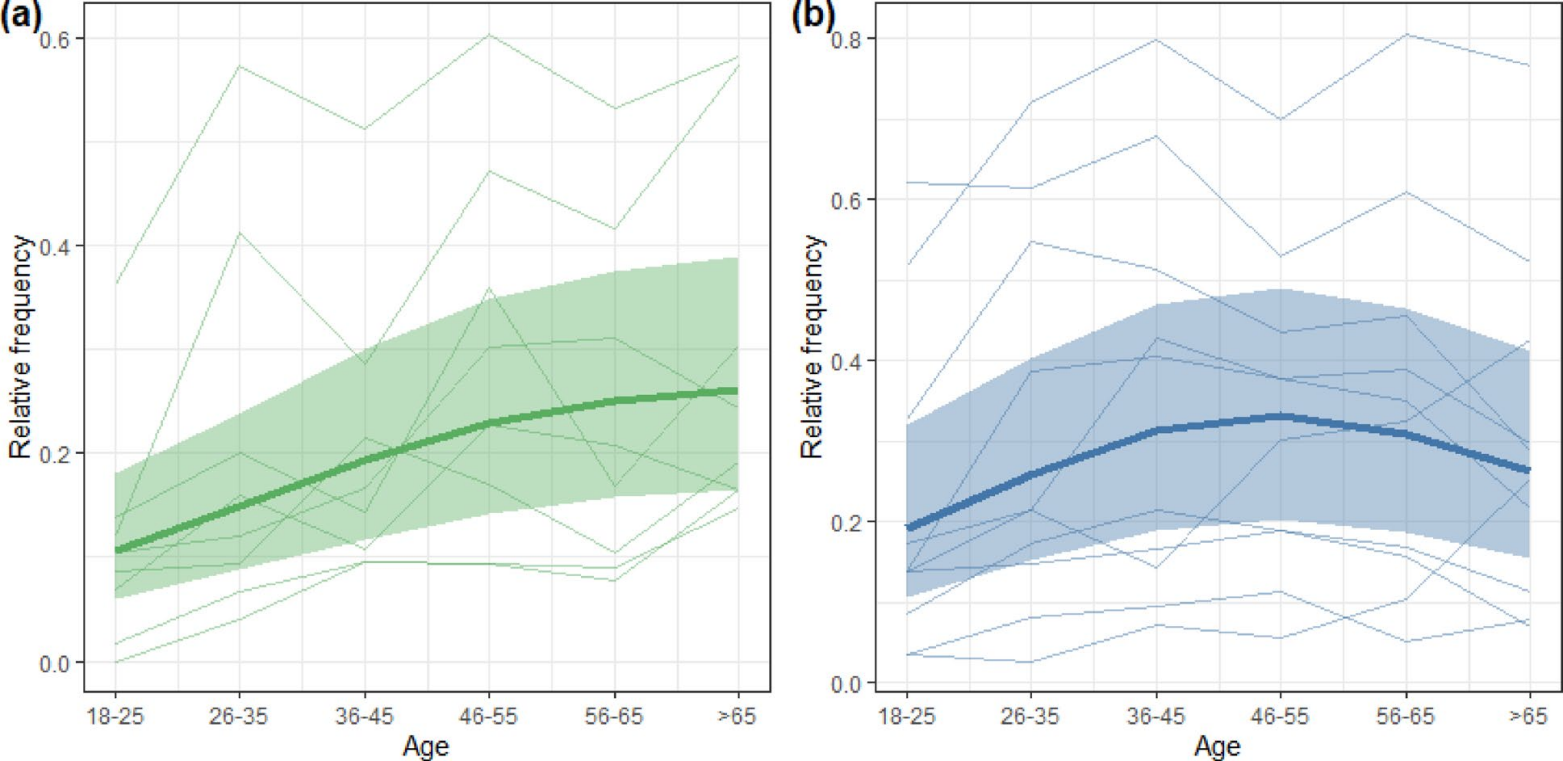
Relative frequency of those eight plant indicator taxa (**a**) and ten bird indicator species (**b**) that belong to the people’s cultural domain for the different age classes. Thin lines show the relative frequency for the different taxa. The thick line is the modelled effect of age on the relative frequency according to a binomial generalized additive mixed model. The ribbon represents the 95% confidence band

The frequency with which participants listed indicator plants increased significantly with age (*L* = 60.0, *df* = 2, *p* < 0.001) with the strongest increase between age classes 18–25 and 46–55 (Fig. 4a). Also, the frequency of indicator birds increased between these age classes 18–25 and 46–55 (*L* = 29.1, *df* = 2, *p* < 0.001) but decreased again beyond this age range (Fig. 4b).

**Fig. 5:**
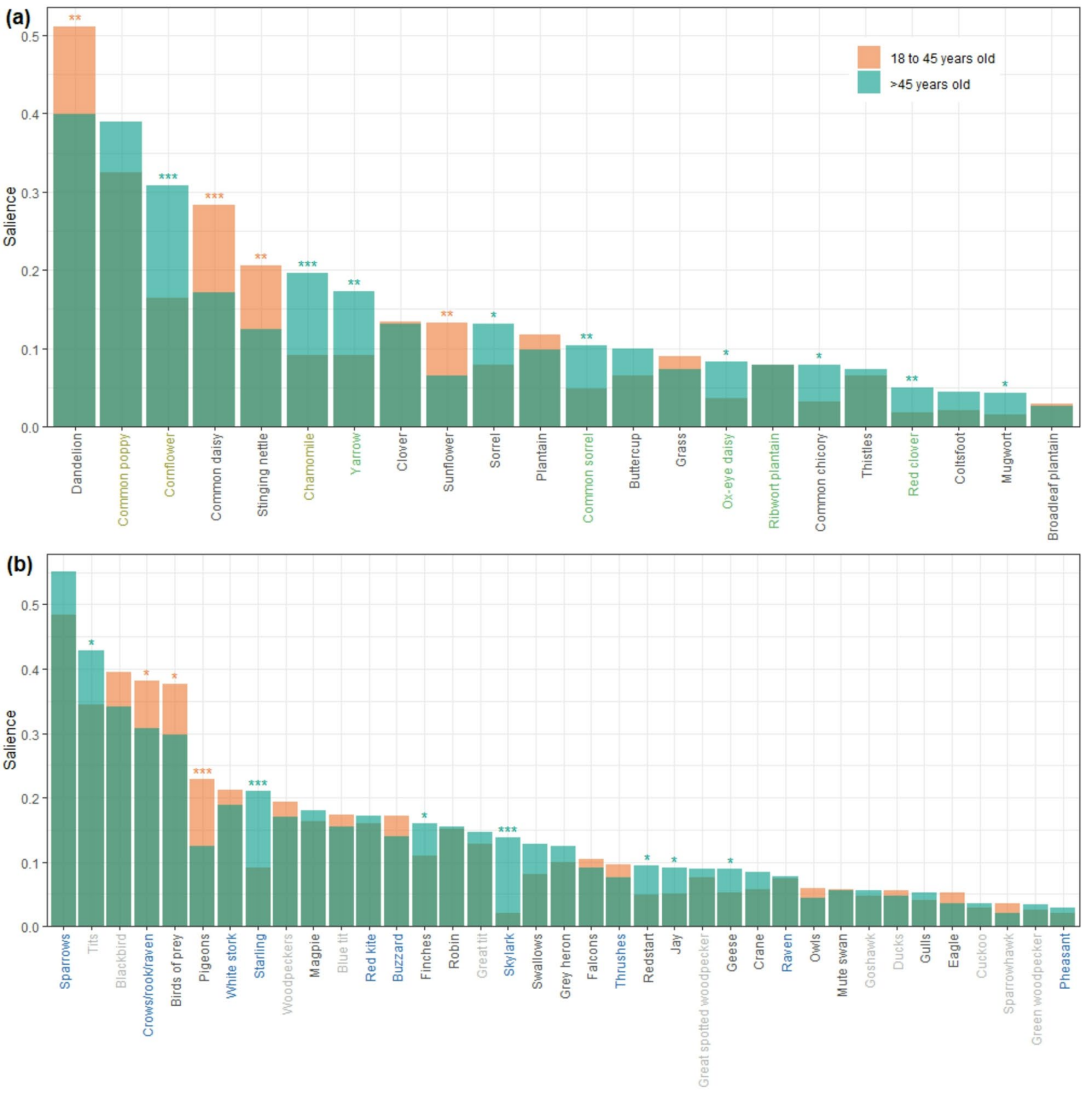
Difference in salience between younger and older participants for all plant (**a**) and bird (**b**) taxa belonging to the people’s cultural domain. Asterisks indicate the level of significance (*** *p* ≤ 0.001, ** *p* ≤ 0.01, * *p* ≤ 0.05) according to a two-sample randomization test. Colours of taxa labels correspond to those in Fig. 2

Nine plant taxa from within the cultural domain had a significantly lower salience among younger (≤ 45 years old) than among older participants (> 45 years old). Six of these were indicator taxa (Fig. 5a). Four taxa had a higher salience among younger participants, all of which were very common generalists (Fig. 5a).

Seven bird taxa from within the cultural domain had a significantly lower salience among younger participants (Fig. 5b). Two of these, Skylark and Starling, were indicator species. Three taxa had a higher salience among younger participants: birds of prey (Accipitriformes), crows/rook/raven (*Corvus* spp.), and pigeons (Columbidae). All three were taxa at an above-species level (genus to order).

## Discussion

In this study, we assessed people’s local knowledge of plant and bird species in a rural region in northwest Saxony, located in eastern Germany. In contrast to previous studies in high-income countries, we conducted a freelisting exercise with adults rather than school children and did not ask for plant and animals in general, but specifically for species occurring in agricultural habitats of the surrounding landscape. As we expected based on previous research, the participants’ local species knowledge was limited, larger for birds than for plants, and increased with age. However, by using cultural domain analysis, our study revealed insights that go beyond those provided by conventional species identification tests.

### Indicator species are largely unknown

Both the cumulative and the individual local species knowledge in northwest Saxony appear to be low. When individuals from other ethnic groups - that more directly depend on wild plant or animal resources - are asked to freelist wild edible plants, they list on average ten or more plant species [67–70]. In contrast, the participants in our survey listed on average six plant taxa - even though we asked for a broader domain, not just edible plants. This finding is in line with previous studies from high-income societies, where people usually list less than ten different taxa [16, 19, 71, 72]. An exception are farmer families from mountain areas [35, 73], who probably make ample use of wild edible or medicinal plants, not only for themselves, but also for tourism and retail associated with a regional identity. Within these families, however, plant knowledge has been gendered. Farm women know more plants than men, and this is largely attributed to the homegardens women have traditionally overseen. A decline in homegardens is associated with a decline in women’s knowledge about plants [35]. Generally, the cumulative number of different plant species listed by low-income, resource-dependent communities ([74]: pp. 233; [75]: pp. 149; [76]: pp. 150–249; [68]: pp. 95) were found to be as high or higher than in our study, although the cultural domains were defined more narrowly (“wild edible plants”, “medicinal plants”) and the sample sizes, which included individuals, were much smaller compared to our study ([74]: pp. 48; [75]: pp. 31; [76]: pp. 18; [68]: pp. 57). Moreover, individuals from low-income, resource-dependent communities list items at the species or even subspecies level [68, 69], whereas individuals from high-income societies often name taxa at a higher taxonomic level [9, 12, 16, 17, 26, 44, 71], our own study being no exception. This comparison shows clearly that wild plants and animals do not play a significant role in people’s life and have largely lost their cultural relevance. The notable absence of several different local names for the same taxon, except for the case of dandelion, might underline the decline in relationships that local people have with the plants in their surroundings. Vernacular names of plants often represent ideas about plants’ associations with their use, which contribute to their naming [77].

The marginal species knowledge documented with our study is in line with numerous tests of people’s species identification skills in other high-income societies [10, 12, 20, 23, 25–27]. In contrast to the studies that used photographs to test the people’s species identification skills, our freelisting approach revealed also which species are part of the people’s cultural domain and which ones are not. Some very common species that were highly salient in our dataset, such as Dandelion, Common daisy, or Blackbird and Magpie, belong to the most frequently listed or recognized plant and bird species in previous European studies (plants: [21, 24, 35, 73]; birds: [10, 14, 22, 23, 25, 28, 37, 40]). However, there are also some interesting discrepancies. For instance, the Common buzzard, which was the bird with the second-lowest identification rate among ten bird species in a species identification test with German school children [22], had a high salience in our data (Fig. 2b). Cornflower, Common poppy, and Yarrow belonged to the most salient plant species in our survey, while they yielded moderate to low identification rates among German school children and their parents [24]. Common grassland plant taxa such as plantain or sorrel were so far not included in species identification tests [24, 31] but frequently listed by our participants. The same is true for some common farmland bird taxa such as Red kite, Crane or Skylark, that were frequently listed by our participants, but so far not included in species identification tests [23, 28, 37, 40]. These discrepancies show that we may get a different picture of the people’s species knowledge if we do not confront them with photographs of selected species, but ask them openly to list what they know, and if we direct their attention to certain habitats, such as agricultural fields and meadows.

Although some of the frequently listed plant and bird taxa belong to the group of indicator species for agricultural landscapes, such as Chamomile, Common poppy, Ribwort plantain or White stork, Starling, and Sparrows (Fig. 2), most people are able to list only very few of the plant and bird species that are characteristic for their surrounding landscape (Fig. 1b, d). The large majority, i.e. 87% of the plant and 76% of the bird indicator species, do not belong to the people’s cultural domain although they are not rare, nor difficult to discover (see Additional file 2). This implies that people do not encounter these species, either because they are rarely out in their surrounding landscape, or, if they are out in the landscape, do not pay attention to these plant and bird species. This is of particular concern, as these indicator species are indicative of the quality of habitats that reflect cultural practices, i.e. types of land use. As such, indicator species are relevant locally, as they connect humans and nature in place. If there is no local knowledge of these plants, the cultural relationship that connects humans with their landscape may be threatened.

We do not believe that this is because people do not value nature or biodiversity. In a related study [78], we asked the same sample of people about their relationship to nature [79]. Their average agreement score on a Likert scale from 1 (“I fully disagree”) to 5 (“I fully agree”) for the statement “I take notice of wildlife wherever I am” was a 4. They also felt that their relationship with nature would be an important part of who they are (mean agreement score: 4.0). This obvious discrepancy between their felt close relationship to nature and their marginal species knowledge is possibly an expression of the shifting baseline syndrome [4, 8]. People are not aware of their estrangement from their local nature and would not miss those many indicator species since they do not know of their existence anyway. Thus, those plant and bird indicator species that are still common in the agricultural landscape of northwest Saxony risk facing the same fate as the many other arable weeds, grassland herbs, and farmland birds that have declined over the last decades in Germany [49–51, 53] and are now extinct or threatened by extinction [80, 81].

### Young people know fewer species

The consistent positive relationship that we observed between freelist length and age is in line with previous studies and reflects a progressive estrangement from nature that may reinforce loss of indicator species. Several other studies from high-income societies have documented a continuously increasing species knowledge during adulthood [11, 23, 28, 29, 34, 36, 37]. While there is ample evidence for increasing species knowledge with age, there are also exceptions. In farmer families in Switzerland, knowledge about local plant species is gained during childhood and adolescence and then does not increase further [35]. For these farmer families, wild plants have a cultural relevance, i.e. they use wild plants as food, fodder, construction material (wood), medicine, ornaments, and other. The local knowledge about which plants to use in which ways is passed on from the parents and other adults to the children and adolescents while they spend time together outside [35]. Particularly in low-income communities that depend more immediately on wild plant and animal resources, learning about plant species and their uses is indispensable to life and therefore an essential part of childhood [29, 67, 76, 82]. During our freelisting interviews, some older participants mentioned that their knowledge came from growing up and living in a village, associated with homegardens and regularly interacting with the non-human world, simply by spending time outdoors. As people spend less time outside, they have fewer opportunities to observe different species and share local knowledge about species with others, keeping local knowledge alive.

The difference in local knowledge of older compared to younger generations in our study suggests that knowledge transmission about the environment and associated cultural practices no longer occurs vertically, from adults to their children, and from one generation to the next. Instead, knowledge transmission is rather unpredictable and often occurs horizontally, within and across peer groups, within and outside of educational settings. This suggests that local species knowledge and associated cultural practices are gradually being lost rather than passed on to the next generation [83–85]. Support for this assumption comes from two rare resurvey studies, in which Gerl et al. [22, 40] repeated surveys first conducted one decade before on German school students’ skills to identify common birds and vertebrates [38, 86]. The school students in the resurveys exhibited 20% and 15% lower identification rates for birds and vertebrates, respectively [22, 40]. Furthermore, there is also comprehensive evidence from longitudinal studies for a growing emotional disconnection of humans in high-income societies from nature [5], that is related to a loss of the cultural practices that connect people to nature. For instance, the analysis of English language cultural products such as fiction books, song lyrics, and movies has shown that references to or depictions of the natural world have markedly decreased during the last 70 century [87–90]. In some Western countries, the interest in nature-based recreation has declined since the 1980s [91]. Therefore, we assume that the observed progressive increase in species knowledge during adulthood largely reflects differences between generations rather than variation across lifetime [92]. Particularly alarming is the low frequency with which young adults listed plant and bird indicator species for agricultural habitats (Fig. 4). Even very common plant indicator species such as Cornflower, Chamomile, Yarrow, or Common sorrel were clearly less salient in the freelists of younger adults (Fig. 5a). The same is true for the common farmland bird indicator species Starling and Skylark (Fig. 5b). In contrast, those taxa more salient among young adults were very common and widespread habitat generalists (Dandelion, Common daisy, Stinging nettle) and taxa at a high taxonomic level, which older adults could usually specify (birds of prey, pigeons, crows). The chance to encounter these generalists in urban or suburban environments is high. Our results suggest therefore that younger adults are less connected to their surrounding agricultural landscape compared to older adults, as their knowledge is not rooted in place and likely not obtained through observations shared between older and younger generations while interacting with the landscape.

### Limitations and future research

With this study, we began our inquiry into people’s local knowledge about the species in their surrounding agricultural landscape. We acknowledge that freelisting is an imperfect method to assess this on its own, and that additionally walking through the fields, asking people questions about plant use [82] and bird behavior, or showing photos, may have provided a more comprehensive account of *what* and *how* people know certain plant and bird species. However, given the scope of our funding and time capacities, we believe that freelisting provided us a solid entry point to explore people’s local biodiversity knowledge. We hope that future research can build upon this to gain more nuanced insights to local knowledge in northwest Saxony or other German regions with complementary methods.

## Conclusions

Our freelisting study revealed that the cumulative knowledge of the people in northwest Saxony still comprises a few plant and a few more bird species that are characteristic for their agricultural landscape. Nevertheless, most of the common indicator species for agricultural habitats in our study region are not part of the people’s cultural domain. The short average list lengths of these indicator species show that many people have lost their local knowledge about biodiversity in their landscape. The still common familiarity with some few of the plant indicator species might result from the fact that they once were of cultural relevance as food or medicinal plants. For instance, Chamomile, Yarrow or Ribwort Plantain are appreciated for their various healing effects [93] and part of common herbal tea mixtures [94]. Common sorrel and dandelion are valued wild foods [95]. Wildflowers such as Common poppy, Cornflower or Ox-eye daisy have obvious ornamental value and decorate the landscape or one’s home. Such cultural purposes could eventually become more popular again through their promotion via environmental education efforts to strengthen local knowledge. Research has shown that usability is a feature that raises interest and awareness in plants [96], which can be best conveyed with first-hand experiences such as growing plants [97], gathering edible plants [98], or crafting with plants [99], and thereby increase plants’ relational value. For instance, Fišer et al. [100] observed a resurgence of foraging for wild plant foods, mushrooms, and medicinal herbs in Slovenia, facilitated by many foraging educators, who communicate via social media and offer many educational opportunities online.

While environmental education certainly has the potential to increase local knowledge by raising the interest in usable wild plants for susceptible people, restoring local knowledge and the cultural relevance of wild plants for the broad public is probably unrealistic. This is even more true for plant and bird species with a less obvious usability. However, teaching the relevance of biodiversity for ecosystem functioning might enhance plants and other organisms’ intrinsic value to people and provide young people with arguments in favour of biodiversity conservation [101]. Research indicates that local knowledge about plants and animals is linked to the ability to reason about ecological systems [15]. Moreover, outdoor education makes biology more attractive to pupils and has been shown to significantly relieve plant awareness disparity [102], while simultaneously being more efficient and sustainable than indoor teaching [103]. This can provide opportunities for young people to interact with and experience ecosystems. The spatial proximity to agricultural habitats in rural or even peri-urban areas may benefit the implementation of outdoor education in schools, counteracting the “extinction of experience.” Arable weeds, grassland plants, and farmland birds represent easy-to-observe study objects and are well suited to teach trophic interactions [104, 105]. Their sensitivity to various degrees of agricultural land-use intensity can be used to raise awareness of human impacts on biodiversity through food consumption [78, 106]. Since local knowledge is important for biodiversity conservation and adapting to change, reconnecting younger people in northwest Saxony to the agricultural systems and the landscape they are a part of is critical.

## Supplementary Information

Below is the link to the electronic supplementary material.

Supplementary Material 1

Supplementary Material 2

Supplementary Material 3

Supplementary Material 4

Supplementary Material 5

Supplementary Material 6

## Data Availability

The dataset supporting the conclusions of this article is available in the BONARES repository, https://doi.org/10.4228/ZALF-HYWK-GS72.

## Declarations

### Statement on AI or LLM tools

No AI or LLM tools were used in this study.
