# Supplementary material for: Young adults in eastern Germany know dandelion and sparrows but few farmland species
Source: J Ethnobiol Ethnomed. 2026 May 14;22:51. doi: 10.1186/s13002-026-00908-2 (PMC13185424; doi:10.1186/s13002-026-00908-2)
Supplement: Supplementary file 1 — Supplementary Material 1 [file 13002_2026_908_MOESM1_ESM.docx]

Additional file 1. Scales used to quantify the particpants’ age, level of education, and monthly net household income.

| **Age (years)** | |  | **Level of education** | |  | **Monthly net household income (€)** | |
| --- | --- | --- | --- | --- | --- | --- | --- |
| 1: | 18 – 25 |  | 1: | “Hauptschulabschluss” (Certificate of Secondary Education) |  | 1: | <1000 |
| 2: | 26 – 35 |  | 2: | “Realschulabschluss” (General Certificate of Secondary Education usually taken after the fifth year of secondary school) or equivalent |  | 2: | 1000 to <1500 |
| 3: | 36 – 45 |  | 3: | “Abitur” (university-entrance diploma acquired at a secondary school in Germany) or equivalent |  | 3: | 1500 to <2500 |
| 4: | 46 – 55 |  | 4: | Bachelor degree or equivalent |  | 4: | 2500 to <3500 |
| 5: | 56 – 65 |  | 5: | University Master degree or equivalent |  | 5: | 3500 to <5000 |
| 6: | >65 |  | 6: | PhD |  | 6: | ≥ 5000 |
