# Supplementary material for: Young adults in eastern Germany know dandelion and sparrows but few farmland species
Source: J Ethnobiol Ethnomed. 2026 May 14;22:51. doi: 10.1186/s13002-026-00908-2 (PMC13185424; doi:10.1186/s13002-026-00908-2)
Supplement: Supplementary file 2 — Supplementary Material 2 [file 13002_2026_908_MOESM2_ESM.docx]

Additional file 2. Plant^1^ and bird^2^ indicator taxa that occur in the study region.

|  | **Taxon** | **English name** | **Frequency ^3^** | **Listed by at least one participant** | **Part of the cultural domain ^4^** |
| --- | --- | --- | --- | --- | --- |
|  |  |  |  |  |  |
| **Grassland plant indicator taxa** | |  |  |  |  |
|  | *Achillea millefolium* L. | Yarrow | 3 | x | x |
|  | *Achillea ptarmica* L. | Sneezewort | 1 |  |  |
|  | *Agrimonia eupatoria* L. | Agrimony | 2 |  |  |
|  | *Anthoxanthum odoratum* L. | Sweet vernalgras | 2 |  |  |
|  | *Armeria* spp. |  | 3 |  |  |
|  | *Bistorta officinalis* Delarbre | Bistort | 1 |  |  |
|  | *Caltha palustris* L. | Marsh marigold | 1 | x |  |
|  | *Campanula* spp. |  | 2 | x |  |
|  | *Cardamine pratensis* L./*amara* L. | Cuckoo flowers | 2 | x |  |
|  | *Carex* spp. |  | 3 |  |  |
|  | *Centaurea* spp. |  | 3 |  |  |
|  | *Cirsium palustre*/*oleraceum* (L.) Scop. | Marsh thistle. | 2 |  |  |
|  | *Dianthus deltoides* L. | Maiden Pink | 2 |  |  |
|  | *Dianthus* spp. | Sweet William | 2 | x |  |
|  | *Filipendula ulmaria* (L.) Maxim. | Meadow sweet | 3 |  |  |
|  | *Galium mollugo* L. | Erect bedstraw | 3 |  |  |
|  | *Galium* spp. (without *G. mollugo* L., *G. verum* L*.*, *G. aparine* L.) | Cangay | 3 |  |  |
|  | *Galium verum* L. | Lady’s bedstraw | 2 |  |  |
|  | *Geranium palustre* L. | Marsh cinquefoil | 1 |  |  |
|  | *Geranium pratense*/*sylvaticum* L. | Cranesbill | 1 | x |  |
|  | *Geum rivale* L. | Water avens | 1 |  |  |
|  | *Hieracium pilosella* L. | Mouse ear | 3 | x |  |
|  | *Hypericum* spp. |  | 3 | x |  |
|  | *Knautia arvensis* (L.) Coult. | Scabious | 1 | x |  |
|  | *Leucanthemum vulgare* Lam. | Ox-eye daisy | 3 | x | x |
|  | *Lotus* spp. |  | 2 | x |  |
|  | *Luzula* spp. |  | 1 |  |  |
|  | *Lychnis flos-cuculi* (L.) Greuter & Burdet |  | 1 | x |  |
|  | *Myosotis* spp. |  | 2 | x |  |
|  | *Plantago lanceolata* L. | Rib grass | 3 | x | x |
|  | *Potentilla* *erecta* (L.) Raeusch. | Cinqefoil | 1 |  |  |
|  | *Potentilla* spp. |  | 3 | x |  |
|  | *Primula* *veris* L. /*elatior* (L.) Hill | Cow slip | 1 | x |  |
|  | *Prunella vulgaris* L. |  | 2 |  |  |
|  | *Ranunculus acris* L. | Meadow buttercup | 3 | x |  |
|  | *Ranunculus* spp. (without *R. repens* L.) | Buttercup | 1 |  |  |
|  | *Rhinanthus* spp. |  | 1 |  |  |
|  | *Rumex acetosa* L. /*thyrsiflorus* Fingerh. |  | 3 | x | x |
|  | *Rumex acetosella* L. |  | 3 |  |  |
|  | *Sanguisorba officinalis* L. | Lesser burnet | 2 |  |  |
|  | *Scabiosa* spp. |  | 1 |  |  |
|  | *Trifolium arvense* L. |  | 2 |  |  |
|  | *Trifolium pratense* L. |  | 3 | x | x |
|  | *Veronica chamaedrys* L. |  | 3 |  |  |
|  | Yellow Asteraceae (without *Taraxacum*; only species without stem leaves, but with just a rosette*; Crepis* spp.., *Hieracium* spp., *Hypochoeris* spp., *Leontodon* spp.) |  | 3 |  |  |
|  |  |  |  |  |  |
| **Arable plant indicator taxa** | |  |  |  |  |
|  | *Aethusa cynapium* L. | Fool’s parsley | 1 |  |  |
|  | *Anagallis arvensis* L. |  | 2 |  |  |
|  | *Anchusa arvensis* (L.) M.Bieb. |  | 3 | x |  |
|  | *Anthemis arvensis* L. | Corn chamomile | 3 |  |  |
|  | *Aphanes arvensis* L. | Parsley | 1 |  |  |
|  | *Arabidopsis thaliana* L. |  | 3 |  |  |
|  | *Buglossoides arvensis* L. |  | 1 |  |  |
|  | *Caucalis* spp. |  | 1 |  |  |
|  | *Centaurea cyanus* L. | Cornflower | 3 | x | x |
|  | *Consolida regalis* L. |  | 1 | x |  |
|  | *Erodium cicutarium* (L.) L'Hér | Common storks bill | 2 |  |  |
|  | *Euphorbia* spp. |  | 3 | x |  |
|  | *Fumaria* spp. | Fumitory herb | 2 |  |  |
|  | *Galeopsis tetrahit* L. | Hemp nettle | 2 |  |  |
|  | *Geranium* spp. |  | 3 | x |  |
|  | *Glebionis segetum* (L.) Fourr. |  | 3 |  |  |
|  | *Gnaphalium uliginosum* L. |  | 1 |  |  |
|  | *Gypsophila muralis* (L.) Ikonn. |  | 1 |  |  |
|  | *Hypochaeris glabra* L. |  | 3 |  |  |
|  | *Kickxia* spp. |  | 1 |  |  |
|  | *Lamium* spp. |  | 3 | x |  |
|  | *Lapsana communis* L. | Nipplewort | 3 |  |  |
|  | *Lathyrus tuberosus* L. | Pea | 2 |  |  |
|  | *Linaria vulgaris* Mill. | Toad flax | 3 |  |  |
|  | *Lythrum* spp. |  | 3 |  |  |
|  | *Matricaria chamomilla* L. | Wild Chamomiles | 3 | x | x |
|  | *Mentha arvensis* L. | Peppermint | 1 |  |  |
|  | *Mutarda arvensis* (L.) D.A.German | Bazzocks | 1 | x |  |
|  | *Myosotis* spp. |  | 3 | x |  |
|  | *Ornithopus perpusillus* L. | Bird’s-foot | 1 |  |  |
|  | *Papaver* spp. | Poppy | 3 | x | x |
|  | *Persicaria lapathifolia* (L.) Delarbre | Pale persicaria | 3 |  |  |
|  | *Ranunculus arvensis* L. | Corn buttercup | 1 |  |  |
|  | *Ranunculus sardous* Crantz | Hairy buttercup | 1 |  |  |
|  | *Raphanus raphanistrum* L. | Wild radish | 3 |  |  |
|  | *Rumex acetosella* L. |  | 3 |  |  |
|  | *Scleranthus annuus* L. | Annual knawel | 3 |  |  |
|  | *Sherardia arvensis* L. | Field madder | 1 |  |  |
|  | *Silene noctiflora* L. | night-flowering catchfly | 1 |  |  |
|  | *Sonchus* spp. |  | 3 |  |  |
|  | *Spergula arvensis* L. | Corn spurry | 1 |  |  |
|  | *Spergularia rubra* (L.) J.Presl & C.Presl | Red sandspurry | 3 |  |  |
|  | *Stachys palustris* L. | Marsh woundwort | 1 |  |  |
|  | *Teesdalia nudicaulis* (L.) W.T.Aiton | Naked Stalked Barnyard Smute | 1 |  |  |
|  | *Thlaspi arvensis* L. | Field pennycress | 3 |  |  |
|  | *Trifolium arvense* L. | Hare's-foot clover | 2 |  |  |
|  | *Valerianella* spp. |  | 3 |  |  |
|  | *Veronica arvensis* L. | Wall speedwell | 1 |  |  |
|  | *Vicia* spp. |  | 3 | x |  |
|  | *Viola arvensis* Murray | Field pansy | 3 |  |  |
|  |  |  |  |  |  |
| **Farmland bird indicator species** | |  |  |  |  |
|  | *Acrocephalus palustris* (Bechstein, 1798) | Marsh Warbler | 2 |  |  |
|  | *Alauda arvensis* (Linnaeus, 1758) | Eurasian Skylark | 3 | x | x |
|  | *Buteo buteo* (Linnaeus, 1758) | Eurasian Buzzard | 3 | x | x |
|  | *Carduelis carduelis* (Linnaeus, 1758) | European Goldfinch | 3 | x |  |
|  | *Ciconia ciconia* |  | 2 | x | x |
|  | *Circus pygargus* |  | 1 | x |  |
|  | *Corvus corax* (Linnaeus, 1758) | White Stork | 3 | x | x |
|  | *Corvus corone* (Linnaeus, 1758) | Carrion Crow | 3 | x | (x) |
|  | *Corvus frugilegus* (Linnaeus, 1758) | Rook | 1 | x | (x) |
|  | *Coturnix coturnix* (Linnaeus, 1758) | Common Quail | 2 | x |  |
|  | *Crex crex* (Linnaeus, 1758) | Corncrake | 1 |  |  |
|  | *Curruca communis (Latham, 1787)* | Common Whitethroat | 3 |  |  |
|  | *Emberiza calandra (Linnaeus, 1758)* | Calandra Lark | 2 |  |  |
|  | *Emberiza citrinella* (Linnaeus, 1758) | Yellowhammer | 3 | x |  |
|  | *Emberiza hortulana* (Linnaeus, 1758) | Ortolan Bunting | 1 |  |  |
|  | *Emberiza schoeniclus* (Linnaeus, 1758) | Reed Bunting | 2 |  |  |
|  | *Gallinago gallinago* (Linnaeus, 1758) | Common Snipe | 1 |  |  |
|  | *Hirundo rustica* (Linnaeus, 1758) | Barn Swallow | 3 | x |  |
|  | *Jynx torquilla* (Linnaeus, 1758) | Eurasian Wryneck | 2 |  |  |
|  | *Lanius collurio* (Linnaeus, 1758) | Red-backed Shrike | 2 | x |  |
|  | *Linaria cannabina* (Linnaeus, 1758) | Common Linnet | 2 | x |  |
|  | *Milvus milvus* (Linnaeus, 1758) | Red Kite | 3 | x | x |
|  | *Motacilla flava* (Linnaeus, 1758) | Western Yellow Wagtail | 2 | x |  |
|  | *Passer domesticus* (Linnaeus, 1758) | House Sparrow | 3 | x | (x) |
|  | *Passer montanus* (Linnaeus, 1758) | Eurasian Tree Sparrow | 2 | x | (x) |
|  | *Phasianus colchicus* (Linnaeus, 1758) | Common Pheasant | 2 | x | x |
|  | *Phoenicurus phoenicurus* (Linnaeus, 1758) | Common Redstart | 3 | x |  |
|  | *Saxicola rubetra* (Linnaeus, 1758) | Whinchat | 1 | x |  |
|  | *Sturnus vulgaris* (Linnaeus, 1758) | Common Starling | 3 | x | x |
|  | *Turdus philomelos* (Brehm, 1831) | Song Thrush | 3 | x | (x) |
|  | *Turdus pilaris* (Linnaeus, 1758) | Fieldfare | 2 | x | (x) |
|  | *Upupa epops* (Linnaeus, 1758) | Common Hoopoe | 1 | x |  |

^1^ Scientific plant names according to Plants of the World Online. Facilitated by the Royal Botanic Gardens, Kew: <https://powo.science.kew.org>; “spp.” means that several species of that genus are meant

^2^ Scientific bird names according to Avilist – The Global Avian Checklist (<https://www.avilist.org/>)

^3^1 – rare (i.e., difficult to discover for the common citizen), 2 – occasional to regular (i.e., with some luck or effort observable for the interested citizen), 3 – common (i.e., easily observable for the common citizen during a walk or bicycle tour)

^4^ Crosses in parentheses indicate that this taxon was only part of the cultural domain as part of a higher-order taxon.
