# Supplementary material for: Young adults in eastern Germany know dandelion and sparrows but few farmland species
Source: J Ethnobiol Ethnomed. 2026 May 14;22:51. doi: 10.1186/s13002-026-00908-2 (PMC13185424; doi:10.1186/s13002-026-00908-2)
Supplement: Supplementary file 3 — Supplementary Material 3 [file 13002_2026_908_MOESM3_ESM.docx]

**Additional file 3**. Reference list of all plant and bird taxa listed by the 463 participants.

**Plant taxa**

| **Scientific name**^1^ | **German vernacular**  **name ^2^** | **English common name ^3^** | **Overlapping**  **higher-order**  **taxon** | **Valid** | **Grassland plant indicator taxon** | **Arable plant indicator taxon** | **Count** | **Relative frequency** | **Mean rank** | **Smith's S** |
| --- | --- | --- | --- | --- | --- | --- | --- | --- | --- | --- |
| *Achillea millefolium* L. | Schafgarbe | Yarrow |  | x | x |  | 102 | 0.2203 | 4.41 | 0.1344 |
| *Aconitum* spp. | Eisenhut |  |  |  |  |  | 1 | 0.0022 | 3.00 | 0.0019 |
| *Aegopodium podagraria* L. | Giersch |  |  | x |  |  | 14 | 0.0302 | 6.07 | 0.0157 |
| *Ajuga reptans* L. | Günsel |  |  | x |  |  | 2 | 0.0043 | 8.00 | 0.0030 |
| *Alliaria petiolate* (M.Bieb.) Cavara & Grande | Knoblauchsrauke |  |  | x |  |  | 2 | 0.0043 | 5.50 | 0.0020 |
| *Allium schoenoprasum* L. | Schnittlauch |  |  |  |  |  | 3 | 0.0065 | 4.33 | 0.0037 |
| *Allium ursinum* L. | Bärlauch |  |  |  |  |  | 19 | 0.0410 | 5.58 | 0.0224 |
| *Alopecurus pratensis Bourg. ex Lange* | Fuchsschwanz |  | Poaceae | x |  |  | 2 | 0.0043 | 4.50 | 0.0023 |
| *Althaea officinalis* L. | Eibisch |  |  |  |  |  | 1 | 0.0022 | 2.00 | 0.0019 |
| *Anchusa* spp. | Ochsenzunge |  |  | x |  | x | 1 | 0.0022 | 21.00 | 0.0002 |
| *Anemone nemorosa* Schangin | Buschwindröschen |  |  |  |  |  | 5 | 0.0108 | 5.80 | 0.0061 |
| *Anethum graveolens* Ucria | Dill |  |  |  |  |  | 1 | 0.0022 | 5.00 | 0.0004 |
| *Angelica sylvestris* L. | Wilde Angelica |  |  | x |  |  | 1 | 0.0022 | 11.00 | 0.0010 |
| *Anthriscus sylvestris* (L.) Hoffm. | Kerbel |  |  | x |  |  | 3 | 0.0065 | 8.33 | 0.0036 |
| *Antirrhinum majus* L. | Löwenmaul |  |  |  |  |  | 1 | 0.0022 | 1.00 | 0.0022 |
| *Apera spica-venti* (L.) P.Beauv. | Windhalm |  | Poaceae | x |  |  | 1 | 0.0022 | 6.00 | 0.0017 |
| *Aquilegia vulgaris* L. | Akelei |  |  | x |  |  | 3 | 0.0065 | 4.00 | 0.0038 |
| *Arctium* spp. | Kletten |  |  | x |  |  | 9 | 0.0194 | 7.78 | 0.0066 |
| *Armoracia rusticana* G.Gaertn., B.Mey. & Scherb. | Meerrettich |  |  | x |  |  | 2 | 0.0043 | 2.50 | 0.0036 |
| *Arnica montana* Hook. | Arnika |  |  |  |  |  | 1 | 0.0022 | 2.00 | 0.0020 |
| *Artemisia absinthium* L. | Wermut |  |  | x |  |  | 1 | 0.0022 | 12.00 | 0.0002 |
| *Artemisia vulgaris* L. | Beifuß | Mugwort |  | x |  |  | 24 | 0.0518 | 5.25 | 0.0293 |
| *Atriplex* spp. | Melde |  |  | x |  |  | 6 | 0.0130 | 6.67 | 0.0052 |
| *Bellis perennis* L. | Gänseblümchen | Common daisy |  | x |  |  | 166 | 0.3585 | 3.88 | 0.2236 |
| *Brassica napus* L. | Wilder Raps |  |  | x |  |  | 9 | 0.0194 | 7.44 | 0.0085 |
| *Calendula* spp. | Ringelblume |  |  |  |  |  | 19 | 0.0410 | 4.37 | 0.0210 |
| *Calluna* *vulgaris* (L.) Hull / *Erica* spp. | Heidekraut |  |  |  |  |  | 2 | 0.0043 | 10.50 | 0.0016 |
| *Caltha palustris* L. | Sumpf-Dotterblume |  |  | x | x |  | 8 | 0.0173 | 5.00 | 0.0091 |
| *Campanula* spp. | Glockenblume |  |  | x | x |  | 13 | 0.0281 | 5.31 | 0.0167 |
| *Capsella bursa-pastoris* (L.) Medik. | Hirtentäschel |  |  | x |  |  | 8 | 0.0173 | 7.13 | 0.0094 |
| *Cardamine pratensis* L. | Wiesen-Schaumkraut |  |  | x | x |  | 1 | 0.0022 | 1.00 | 0.0022 |
| *Carlina acaulis* L. | Silberdistel |  |  |  |  |  | 2 | 0.0043 | 12.00 | 0.0012 |
| *Carum carvi* L. | Wiesen-Kümmel |  |  | x |  |  | 1 | 0.0022 | 4.00 | 0.0018 |
| *Centaurea cyanus* L. | Kornblume | Cornflower |  | x |  | x | 186 | 0.4017 | 4.51 | 0.2411 |
| *Centaurium erythraea* Rafn | Tausendgüldenkraut |  |  | x |  |  | 1 | 0.0022 | 5.00 | 0.0017 |
| *Chaerophyllum temulum* L. | Kälberkropf |  |  | x |  |  | 1 | 0.0022 | 10.00 | 0.0011 |
| *Chelidonium majus* L. | Schöllkraut |  |  |  |  |  | 1 | 0.0022 | 8.00 | 0.0008 |
| *Cichorium intybus* L. | Wegwarte | Common chicory |  | x |  |  | 43 | 0.0929 | 4.88 | 0.0560 |
| *Cirsium arvense* (L.) Scop. | Acker-Kratzdistel |  | *Cirsium* spp. | x |  |  | 1 | 0.0022 | 9.00 | 0.0007 |
| *Cirsium* spp. | Disteln | Thistles |  | x |  |  | 75 | 0.1620 | 6.00 | 0.0688 |
| *Cirsium vulgare* (Savi) Ten. | Gemeine Distel |  | *Cirsium* spp. | x |  |  | 1 | 0.0022 | 16.00 | 0.0005 |
| *Colchicum autumnale* L. | Herbst-Zeitlose |  |  | x |  |  | 2 | 0.0043 | 6.50 | 0.0009 |
| *Consolida regalis* Gray | Rittersporn |  |  | x |  | x | 1 | 0.0022 | 2.00 | 0.0018 |
| *Convallaria majalis* L. | Maiglöckchen |  |  |  |  |  | 7 | 0.0151 | 6.14 | 0.0065 |
| *Convolvulus arvensis* L. | Acker-Winde |  |  | x |  |  | 3 | 0.0065 | 12.67 | 0.0016 |
| *Cosmos bipinnatus* Cav. | Cosmea |  |  |  |  |  | 2 | 0.0043 | 2.50 | 0.0036 |
| *Crocus* spp. | Krokus |  |  |  |  |  | 11 | 0.0238 | 6.18 | 0.0103 |
| *Dactylis glomerata* L. | Knäulgras |  | Poaceae | x |  |  | 4 | 0.0086 | 9.75 | 0.0038 |
| *Daucus carota* L. | Wilde Möhre |  |  | x |  |  | 14 | 0.0302 | 5.71 | 0.0156 |
| *Dianthus* spp. | Nelken |  |  | x | x |  | 3 | 0.0065 | 6.67 | 0.0046 |
| *Digitalis purpurea* L. | Fingerhut |  |  |  |  |  | 5 | 0.0108 | 4.60 | 0.0060 |
| *Diplotaxis tenuifolia* (L.) DC. | Wilde Rauke |  |  | x |  |  | 4 | 0.0086 | 4.50 | 0.0047 |
| *Dipsacus* spp. | Karden |  |  | x |  |  | 1 | 0.0022 | 10.00 | 0.0009 |
| *Echinops* spp. | Kugeldistel |  |  | x |  |  | 1 | 0.0022 | 9.00 | 0.0002 |
| *Echium vulgare* L. | Natternkopf |  |  | x |  |  | 3 | 0.0065 | 4.67 | 0.0050 |
| *Elymus repens* (L.) Gould | Quecke |  | Poaceae | x |  |  | 3 | 0.0065 | 13.33 | 0.0021 |
| *Equisetum arvense* L. | Acker-Schachtelhalm |  | *Equisetum* spp. | x |  |  | 12 | 0.0259 | 8.17 | 0.0119 |
| *Equisetum* spp. | Schachtelhalm |  |  | x |  |  | 17 | 0.0367 | 7.59 | 0.0194 |
| *Eranthis hyemalis* (L.) Salisb*.* | Winterling |  |  |  |  |  | 1 | 0.0022 | 8.00 | 0.0008 |
| *Erica* spp. | Erica |  |  |  |  |  | 1 | 0.0022 | 8.00 | 0.0013 |
| *Euphorbia* spp. | Wolfsmilch |  |  | x |  | x | 2 | 0.0043 | 4.50 | 0.0022 |
| *Fallopia japonica* (Houtt.) Ronse Decr. | Japan-Knöterich |  | Polygonaceae | x |  |  | 4 | 0.0086 | 9.25 | 0.0043 |
| *Fragaria vesca* L. | (Wald-)Erdbeere |  |  |  |  |  | 6 | 0.0130 | 5.33 | 0.0062 |
| *Galanthus nivalis* L. | Schneeglöckchen |  |  |  |  |  | 15 | 0.0324 | 5.80 | 0.0135 |
| *Galinsoga parviflora* Cav. | Kleinblütiges Franzosenkraut |  | *Galinsoga* spp. | x |  |  | 1 | 0.0022 | 15.00 | 0.0008 |
| Galinsoga spp. | Franzosenkraut |  |  | x |  |  | 3 | 0.0065 | 10.33 | 0.0021 |
| *Galium aparine* L. | Klebkraut |  |  | x |  |  | 2 | 0.0043 | 12.00 | 0.0016 |
| *Galium odoratum* (L.) Scop. | Waldmeister |  |  |  |  |  | 1 | 0.0022 | 7.00 | 0.0005 |
| *Genista* spp./*Cytisus* spp. | Ginster |  |  | x |  |  | 3 | 0.0065 | 3.00 | 0.0037 |
| *Gentiana* spp. | Enzian |  |  |  |  |  | 1 | 0.0022 | 2.00 | 0.0011 |
| *Geranium pratense* L. | Wiesen-Storchschnabel |  | *Geranium* spp. | x | x |  | 1 | 0.0022 | 9.00 | 0.0014 |
| *Geranium* spp. | Storchschnabel |  |  | x |  | x | 3 | 0.0065 | 9.67 | 0.0028 |
| *Glechoma hederacea* L. | Gundermann |  |  | x |  |  | 4 | 0.0086 | 9.75 | 0.0032 |
| *Helianthus annuus* L. | Sonnenblume | Sunflower |  | x |  |  | 75 | 0.1620 | 4.31 | 0.0964 |
| *Helictotrichon pubescens* (Huds.) Pilg. | Wilder Hafer |  | Poaceae | x |  |  | 4 | 0.0086 | 9.50 | 0.0040 |
| *Heracleum mantegazzianum Sommier & Levier* | Riesen-Bärenklau |  | *Heracleum* spp. | x |  |  | 1 | 0.0022 | 10.00 | 0.0011 |
| *Heracleum sphondylium* L. | Wiesen-Bärenklau |  | *Heracleum* spp. | x |  |  | 2 | 0.0043 | 5.50 | 0.0033 |
| *Heracleum* spp. | Bärenklau |  |  | x |  |  | 5 | 0.0108 | 4.20 | 0.0068 |
| *Hieracium* spp. | Habichtskraut |  |  | x | x |  | 1 | 0.0022 | 4.00 | 0.0014 |
| *Hordeum murinum* L. | Mäuse-Gerste |  | Poaceae | x |  |  | 1 | 0.0022 | 8.00 | 0.0006 |
| *Humulus lupulus* L. | Hopfen |  |  |  |  |  | 1 | 0.0022 | 4.00 | 0.0005 |
| *Hyacinthus orientalis* L. | Hyazinthe |  |  |  |  |  | 4 | 0.0086 | 6.75 | 0.0043 |
| *Hypericum* spp. | Johanniskraut |  |  | x | x |  | 4 | 0.0086 | 4.50 | 0.0054 |
| *Impatiens glandulifera* Arn. | Drüsiges Springkraut |  |  | x |  |  | 3 | 0.0065 | 8.67 | 0.0037 |
| *Impatiens* spp. | Springkraut (gelb) |  |  |  |  |  | 1 | 0.0022 | 23.00 | 0.0001 |
| *Juncus* spp. | Binsen |  |  | x |  |  | 1 | 0.0022 | 6.00 | 0.0004 |
| *Knautia arvensis* (L.) Coult. | Acker-Witwenblume |  |  | x | x |  | 1 | 0.0022 | 9.00 | 0.0004 |
| *Lamium* spp. | Taubnessel |  |  | x |  | x | 17 | 0.0367 | 6.94 | 0.0164 |
| *Lathyrus pratensis* L. | Wiesen-Platterbse |  |  | x |  |  | 1 | 0.0022 | 20.00 | 0.0004 |
| *Lavandula angustifolia* Mill. | Lavendel |  |  |  |  |  | 12 | 0.0259 | 3.25 | 0.0151 |
| *Lepidium sativum* L. | Kresse |  |  |  |  |  | 1 | 0.0022 | 3.00 | 0.0011 |
| *Leucanthemum vulgare* Lam. | Margerite | Ox-eye daisy |  | x | x |  | 44 | 0.0950 | 4.36 | 0.0606 |
| *Lilium* spp. | Lilien |  |  |  |  |  | 4 | 0.0086 | 4.25 | 0.0042 |
| *Linum usitatissimum* L. | Flachs |  |  |  |  |  | 1 | 0.0022 | 10.00 | 0.0007 |
| *Lolium perenne* L. | Weidelgras |  | Poaceae | x |  |  | 1 | 0.0022 | 13.00 | 0.0009 |
| *Lolium pratense (Huds.) Darbysh.* | Wiesen-Schwingel |  | Poaceae | x |  |  | 2 | 0.0043 | 7.00 | 0.0031 |
| *Lotus* spp. | Hornklee |  |  | x | x |  | 2 | 0.0043 | 16.50 | 0.0013 |
| *Lupinus polyphyllus* Lindl. | Lupine |  |  | x |  |  | 11 | 0.0238 | 5.18 | 0.0127 |
| *Lychnis flos-cuculi* L. | Kuckucksblume |  |  | x | x |  | 2 | 0.0043 | 3.50 | 0.0035 |
| *Malva* spp. | Malve |  |  | x |  |  | 8 | 0.0173 | 7.88 | 0.0075 |
| *Matricaria* spp./ *Tripleurospermum* spp. | Kamille | Chamomile |  | x |  | x | 97 | 0.2095 | 3.28 | 0.1463 |
| *Medicago lupulina* L. / *Trifolium dubium* Sibth. / *Tifolium campestre* C.C.Gmel | Gelber Klee |  |  | x |  |  | 3 | 0.0065 | 4.33 | 0.0039 |
| *Medicago sativa* L. | Luzerne |  |  | x |  |  | 3 | 0.0065 | 6.00 | 0.0024 |
| *Melissa officinalis* L. | (Zitronen-)Melisse |  |  |  |  |  | 3 | 0.0065 | 6.33 | 0.0022 |
| *Mentha piperita* L. | Pfeffer-Minze |  |  |  |  |  | 6 | 0.0130 | 3.83 | 0.0047 |
| *Mentha* spp. | Minze |  |  | x |  |  | 6 | 0.0130 | 6.17 | 0.0071 |
| *Muscari armeniacum* H.J.Veitch | Traubenhyazinthe |  |  |  |  |  | 1 | 0.0022 | 7.00 | 0.0010 |
| *Myosotis* spp. | Vergissmeinnicht |  |  | x | x | x | 4 | 0.0086 | 7.75 | 0.0029 |
| *Narcissus pseudonarcissus* L. | Osterglocke/Narzisse |  |  |  |  |  | 10 | 0.0216 | 4.90 | 0.0096 |
| *Nasturtium officinale* W.T.Aiton | Brunnenkresse |  |  |  |  |  | 1 | 0.0022 | 4.00 | 0.0011 |
| *Nepeta cataria* L. | Katzenminze |  |  | x |  |  | 1 | 0.0022 | 8.00 | 0.0003 |
| *Nymphaea alba* L. | Seerose |  |  |  |  |  | 1 | 0.0022 | 4.00 | 0.0009 |
| Orchidaceae | Orchideen |  |  | x |  |  | 5 | 0.0108 | 3.20 | 0.0088 |
| *Origanum majorana* L. | Majoran |  |  |  |  |  | 2 | 0.0043 | 3.50 | 0.0018 |
| *Oxalis* spp. | Sauerklee |  |  | x |  |  | 1 | 0.0022 | 5.00 | 0.0009 |
| *Paederia lanuginose* Wall. | Käsekraut |  |  |  |  |  | 1 | 0.0022 | 6.00 | 0.0004 |
| Panicoideae | Wilde Hirse |  |  | x |  |  | 2 | 0.0043 | 12.50 | 0.0011 |
| *Papaver rhoeas* L. | Mohn(-blume) | Common poppy |  | x |  | x | 247 | 0.5335 | 3.76 | 0.3587 |
| *Petroselinum crispum (Mill.) Fuss* | Petersilie |  |  |  |  |  | 3 | 0.0065 | 1.67 | 0.0059 |
| *Phacelia tanacetifolia* Benth. | Phacelia |  |  |  |  |  | 4 | 0.0086 | 4.00 | 0.0048 |
| *Phleum pratense* L. | Lieschgras |  | Poaceae | x |  |  | 1 | 0.0022 | 10.00 | 0.0005 |
| *Phlox paniculata* L. | Phlox |  |  |  |  |  | 1 | 0.0022 | 3.00 | 0.0014 |
| *Phragmites australis* (Cav.) Trin. ex Steud. | Schilfgras |  | Poaceae | x |  |  | 3 | 0.0065 | 7.00 | 0.0017 |
| *Phyteuma* spp. | Teufelskralle |  |  | x |  |  | 1 | 0.0022 | 5.00 | 0.0012 |
| *Pimpinella* spp. | Pimpinelle |  |  | x |  |  | 1 | 0.0022 | 18.00 | 0.0006 |
| *Plantago lanceolata* L. | Spitz-Wegerich | Ribwort plantain | *Plantago* spp. | x | x |  | 69 | 0.1490 | 5.49 | 0.0789 |
| *Plantago major* L. | Breit-Wegerich | Broadleaf plantain | *Plantago* spp. | x |  |  | 24 | 0.0518 | 6.13 | 0.0278 |
| *Plantago* spp. | Wegerich | Plantain |  | x |  |  | 76 | 0.1641 | 4.36 | 0.1066 |
| *Poa* spp. | Rispengras |  | Poaceae | x |  |  | 1 | 0.0022 | 9.00 | 0.0002 |
| Poaceae | Gräser | Grass |  | x |  |  | 81 | 0.1749 | 5.58 | 0.0807 |
| Polygonaceae | Knöterich |  |  | x |  |  | 6 | 0.0130 | 6.67 | 0.0083 |
| Polypodiaceae | Farne |  |  |  |  |  | 6 | 0.0130 | 6.33 | 0.0041 |
| *Portulaca oleracea* L. | Portulak |  |  | x |  |  | 1 | 0.0022 | 7.00 | 0.0005 |
| *Potentilla anserina* L. | Gänse-Fingerkraut |  | *Potentilla* spp. | x | x |  | 1 | 0.0022 | 3.00 | 0.0017 |
| *Potentilla* spp. | Fingerkraut |  |  | x | x |  | 2 | 0.0043 | 8.50 | 0.0024 |
| *Primula veris* L. | Schlüsselblume |  |  | x | x |  | 11 | 0.0238 | 7.09 | 0.0124 |
| *Pulmonaria* spp. | Lungenkraut |  |  |  |  |  | 1 | 0.0022 | 7.00 | 0.0016 |
| *Ranunculus acris* L. | Scharfer Hahnenfuß |  | *Ranunculus* spp. | x | x |  | 1 | 0.0022 | 3.00 | 0.0020 |
| *Ranunculus* spp. | Butterblume | Buttercup |  | x |  |  | 58 | 0.1253 | 3.97 | 0.0828 |
| *Rubus fruticosus* L. | Brombeere |  |  | x |  |  | 19 | 0.0410 | 5.05 | 0.0223 |
| *Rubus idaeus* L. | Himbeere |  |  |  |  |  | 10 | 0.0216 | 5.50 | 0.0103 |
| *Rumex acetosa* L. / *thyrsiflorus* Fingerh. | Sauer-Ampfer | Common sorrel | *Rumex* spp. | x | x |  | 72 | 0.1555 | 5.46 | 0.0773 |
| *Rumex sanguineus* L. | Blut-Ampfer |  | *Rumex* spp. |  |  |  | 1 | 0.0022 | 6.00 | 0.0015 |
| *Rumex* spp. | Ampfer | Sorrel |  | x |  |  | 79 | 0.1706 | 4.46 | 0.1065 |
| *Salvia pratensis* L. | Salbei |  |  | x |  |  | 10 | 0.0216 | 3.40 | 0.0138 |
| *Sedum acre* L. | Mauerpfeffer |  |  | x |  |  | 1 | 0.0022 | 11.00 | 0.0002 |
| *Senecio jacobaea* Gaertn. | Jakobskraut/Kreuzkraut |  |  | x |  |  | 3 | 0.0065 | 10.67 | 0.0033 |
| *Silene* spp. | Lichtnelke |  |  | x |  |  | 1 | 0.0022 | 8.00 | 0.0014 |
| *Sinapis* spp. | Wilder Senf |  |  | x |  | x | 3 | 0.0065 | 11.33 | 0.0011 |
| Solanaceae | Nachtschattengewächse |  |  |  |  |  | 1 | 0.0022 | 2.00 | 0.0019 |
| *Solenostemon scutellarioides* (L.) Codd | Buntnessel |  |  |  |  |  | 1 | 0.0022 | 14.00 | 0.0003 |
| *Solidago* spp. | Goldrute |  |  | x |  |  | 9 | 0.0194 | 4.22 | 0.0113 |
| *Stellaria media* (L.) Vill. | Vogel-Miere |  |  | x |  |  | 6 | 0.0130 | 9.83 | 0.0052 |
| *Sternbergia lutea* (L.) Ker Gawl. ex Spreng. | Gewitterblume |  |  |  |  |  | 2 | 0.0043 | 8.00 | 0.0012 |
| *Tanacetum vulgare* L. | Rainfarn |  |  | x |  |  | 5 | 0.0108 | 5.60 | 0.0064 |
| *Taraxacum* spp. | Löwenzahn, Speckblume, Hundeblume | Dandelion |  | x |  |  | 300 | 0.6479 | 3.25 | 0.4510 |
| *Thunbergia alata* Bojer ex Sims | Schwarzäugige Susanne |  |  |  |  |  | 1 | 0.0022 | 3.00 | 0.0007 |
| *Thymus* spp. | Thymian |  |  | x | x |  | 6 | 0.0130 | 5.17 | 0.0051 |
| *Trifolium pratense* L. | Rot-Klee | Red clover | *Trifolium* spp. | x | x |  | 40 | 0.0864 | 8.15 | 0.0349 |
| *Trifolium repens* L. | Weiß-Klee |  | *Trifolium* spp. | x |  |  | 20 | 0.0432 | 9.40 | 0.0143 |
| *Trifolium* spp. | Klee | Clover |  | x |  |  | 118 | 0.2549 | 5.37 | 0.1324 |
| *Tripleurospermum perforatum* (Mérat) Wagenitz | Unechte Kamille |  | *Matricaria* spp./ *Tripleurospermum* spp. | x |  |  | 1 | 0.0022 | 5.00 | 0.0013 |
| *Tulipa* spp. | Tulpe |  |  |  |  |  | 10 | 0.0216 | 5.50 | 0.0106 |
| *Tussilago farfara* L. | Huflattich | Coltsfoot |  | x |  |  | 28 | 0.0605 | 5.29 | 0.0331 |
| *Urtica dioica* L. | Brennnessel | Stinging nettle |  | x |  |  | 129 | 0.2786 | 4.32 | 0.1626 |
| *Vaccinium myrtillus* L. | Heidelbeere/Blaubeere |  |  |  |  |  | 6 | 0.0130 | 4.83 | 0.0072 |
| *Verbascum* spp. | Königskerze |  |  | x |  |  | 6 | 0.0130 | 8.17 | 0.0044 |
| *Vicia* spp. | Wicke |  |  | x |  | x | 6 | 0.0130 | 4.17 | 0.0079 |
| *Vinca minor* L. | Immergrün |  |  |  |  |  | 1 | 0.0022 | 6.00 | 0.0013 |
| *Viola* spp. | Stiefmütterchen/Veilchen |  |  | x |  |  | 15 | 0.0324 | 6.40 | 0.0155 |
| - | Feuerblume |  |  |  |  |  | 1 | 0.0022 | 2.00 | 0.0014 |
| - | Hasenpfötchen |  |  |  |  |  | 1 | 0.0022 | 9.00 | 0.0002 |
| - | Kopfblume |  |  |  |  |  | 1 | 0.0022 | 2.00 | 0.0016 |
| - | Morgenstern |  |  |  |  |  | 1 | 0.0022 | 3.00 | 0.0007 |
| - | Nelken (Frühblüher) |  |  |  |  |  | 1 | 0.0022 | 8.00 | 0.0009 |
| - | Salzgras |  |  |  |  |  | 1 | 0.0022 | 7.00 | 0.0003 |
| - | Schäfchenblume (Gras) |  |  |  |  |  | 1 | 0.0022 | 6.00 | 0.0010 |
| - | Schokoladenblume |  |  |  |  |  | 1 | 0.0022 | 6.00 | 0.0004 |

^1^ Scientific plant names according to Plants of the World Online. Facilitated by the Royal Botanic Gardens, Kew: <https://powo.science.kew.org>; “spp.” means that several species of that genus are meant

^2^ Name used by most of the participants

^3^ Only for those taxa in the cultural domain, i.e. with a relative frequency ≥ 0.05.

Bird taxa

| **Scientific name ^1^** | **German vernacular name ^2^** | **English comon name ^3^** | **Overlapping higher-order taxon** | **Overlapping even- higher-order taxon** | **Valid** | **Farmland bird indicator species** | **Count** | **Relative frequency** | **Mean rank** | **Smith's S** |
| --- | --- | --- | --- | --- | --- | --- | --- | --- | --- | --- |
| *Acanthis flammea* (Linnaeus, 1758) | Birkenzeisig |  | Fringillidae |  |  |  | 1 | 0.0022 | 24.000 | 0.0005 |
| *Accipiter gentilis* (Linnaeus, 1758) | Habicht | Goshawk |  | Accipitriformes |  |  | 48 | 0.1037 | 7.958 | 0.0513 |
| *Accipiter nisus* (Linnaeus, 1758) | Sperber | Sparrowhawk |  | Accipitriformes |  |  | 24 | 0.0518 | 8.375 | 0.0271 |
| Accipitridae | Geier |  |  | Accipitriformes |  |  | 2 | 0.0043 | 5.000 | 0.0027 |
| Accipitriformes | Greifvögel | Birds of prey |  |  | x |  | 248 | 0.5356 | 5.819 | 0.3341 |
| *Aegithalos caudatus* (Linnaeus, 1758) | Schwanzmeise |  | Paridae |  |  |  | 6 | 0.0130 | 9.333 | 0.0080 |
| *Alauda arvensis* (Linnaeus, 1758) | (Feld-)Lerche | Skylark |  |  | x | x | 50 | 0.1080 | 5.640 | 0.0821 |
| *Alcedo atthis* (Linnaeus, 1758) | Eisvogel |  |  |  | x |  | 21 | 0.0454 | 9.429 | 0.0241 |
| *Alopochen aegyptiacus* (Linnaeus, 1766) | Nilgans |  |  |  | x |  | 9 | 0.0194 | 18.000 | 0.0050 |
| *Anas platyrhynchos* (Linnaeus, 1758) | Stockente |  | Anatinae |  |  |  | 12 | 0.0259 | 13.417 | 0.0093 |
| Anatinae | Enten | Ducks |  |  |  |  | 77 | 0.1663 | 10.922 | 0.0507 |
| *Anser answer* (Linnaeus, 1758) | Graugans |  | Anserini |  | x |  | 15 | 0.0324 | 10.533 | 0.0142 |
| *Anser fabalis* (Latham, 1787) | Saatgans |  | Anserini |  | x |  | 2 | 0.0043 | 18.000 | 0.0011 |
| Anserini | Wildgänse | Geese |  |  | x |  | 86 | 0.1857 | 10.814 | 0.0719 |
| *Anthus pratensis* (Linnaeus, 1758) | Wiesenpieper |  |  |  | x | x | 1 | 0.0022 | 8.000 | 0.0013 |
| *Anthus trivialis* (Linnaeus, 1758) | Baumpieper |  |  |  | x |  | 1 | 0.0022 | 12.000 | 0.0012 |
| *Apus apus* (Linnaeus, 1758) | Mauersegler |  |  |  |  |  | 16 | 0.0346 | 15.250 | 0.0097 |
| *Aquila chrysaetos* (Linnaeus, 1758) | Steinadler |  | Aquila chrysaetos/ Pandion haliaetus/ Haliaeetus albicilla | Accipitriformes |  |  | 1 | 0.0022 | 11.000 | 0.0008 |
| *Ardea alba* (Linnaeus, 1758) | Silberreiher |  |  |  | x |  | 14 | 0.0302 | 11.786 | 0.0134 |
| *Ardea cinerea* (Linnaeus, 1758) | (Fisch-)Reiher | Grey heron |  |  | x |  | 131 | 0.2829 | 9.779 | 0.1119 |
| *Asio otus* (Linnaeus, 1758) | Waldohreule |  | Strigiformes |  |  |  | 4 | 0.0086 | 12.750 | 0.0044 |
| *Aythya farina* (Linnaeus, 1758) | Tafelente |  | Anatinae |  |  |  | 1 | 0.0022 | 16.000 | 0.0008 |
| *Botaurus stellaris* (Linnaeus, 1758) | Rohrdommel |  |  |  |  |  | 1 | 0.0022 | 12.000 | 0.0013 |
| *Branta leucopsis* (Bechstein, 1803) | Nonnengans |  | Anserini |  | x |  | 1 | 0.0022 | 5.000 | 0.0018 |
| *Bubo bubo* (Linnaeus, 1758) | Uhu |  | Strigiformes |  |  |  | 14 | 0.0302 | 10.571 | 0.0101 |
| *Buteo buteo* (Linnaeus, 1758) | (Mäuse-)Bussard | Buzzard |  | Accipitriformes | x | x | 143 | 0.3089 | 7.860 | 0.1542 |
| *Carduelis carduelis* (Linnaeus, 1758) | Stieglitz |  | Fringillidae |  | x | x | 13 | 0.0281 | 8.308 | 0.0153 |
| *Chloris chloris* (Linnaeus, 1758) | Grünfink |  | Fringillidae |  | x |  | 17 | 0.0367 | 11.824 | 0.0156 |
| *Ciconia Ciconia* (Linnaeus, 1758) | Storch | White stork |  |  | x | x | 194 | 0.4190 | 7.845 | 0.1989 |
| *Ciconia nigra* (Linnaeus, 1758) | Schwarzstorch |  |  |  |  |  | 1 | 0.0022 | 5.000 | 0.0004 |
| *Circus aeruginosus* (Linnaeus, 1758) | Rohrweihe |  |  | Accipitriformes | x |  | 3 | 0.0065 | 14.000 | 0.0015 |
| *Circus cyaneus* (Linnaeus, 1758) | Kornweihe |  |  | Accipitriformes | x |  | 3 | 0.0065 | 5.333 | 0.0047 |
| *Circus pygargus* (Linnaeus, 1758) | Wiesenweihe |  |  | Accipitriformes | x | x | 2 | 0.0043 | 6.000 | 0.0027 |
| *Coccothraustes coccothraustes* (Linnaeus, 1758) | Kernbeißer |  | Fringillidae |  |  |  | 1 | 0.0022 | 21.000 | 0.0003 |
| *Coloeus monedula* (Linnaeus, 1758) | Dohle |  | Corvus spp. |  | x |  | 14 | 0.0302 | 12.714 | 0.0134 |
| *Columba livia* f. *domestica* (Gmelin, JF, 1789) | Stadttaube |  | Columbidae |  |  |  | 3 | 0.0065 | 14.667 | 0.0015 |
| *Columba oenas* (Linnaeus, 1758) | Hohltaube |  | Columbidae |  |  |  | 2 | 0.0043 | 11.000 | 0.0017 |
| *Columba palumbus* (Linnaeus, 1758) | Ringeltaube |  | Columbidae |  | x |  | 12 | 0.0259 | 15.167 | 0.0084 |
| Columbidae | Tauben | Pigeons |  |  | x |  | 169 | 0.3650 | 7.118 | 0.1734 |
| *Corvus corax* (Linnaeus, 1758) | (Kolk-)Rabe | Raven | Corvus spp. |  | x | x | 69 | 0.1490 | 8.522 | 0.0757 |
| *Corvus cornix* (Linnaeus, 1758) | Nebelkrähe |  | Corvus spp. |  | x | x | 11 | 0.0238 | 9.909 | 0.0125 |
| *Corvus corone* (Linnaeus, 1758) | Rabenkrähe |  | Corvus spp. |  | x | x | 1 | 0.0022 | 20.000 | 0.0002 |
| *Corvus frugilegus* (Linnaeus, 1758) | Saatkrähe |  | Corvus spp. |  | x | x | 14 | 0.0302 | 11.429 | 0.0163 |
| *Corvus* spp. | Krähen/Raben | Crows/rook/raven |  |  | x | x | 274 | 0.5918 | 6.106 | 0.3421 |
| *Coturnix coturnix* (Linnaeus, 1758) | Wachtel |  |  |  | x | x | 3 | 0.0065 | 14.000 | 0.0026 |
| *Cuculus canorus* (Linnaeus, 1758) | Kuckuck | Cuckoo |  |  |  |  | 33 | 0.0713 | 9.030 | 0.0330 |
| *Cyanistes caeruleus* (Linnaeus, 1758) | Blaumeise | Blue tit | Paridae |  |  |  | 122 | 0.2635 | 6.443 | 0.1629 |
| *Cygnus atratus* (Latham, 1790) | Trauerschwan |  |  |  |  |  | 1 | 0.0022 | 25.000 | 0.0005 |
| *Cygnus olor* (J.F.Gmelin, 1789) | Schwan | Mute swan |  |  | x |  | 77 | 0.1663 | 10.987 | 0.0570 |
| *Delichon urbicum* (Linnaeus, 1758) | Mehlschwalbe |  | Hirundinidae |  | x |  | 5 | 0.0108 | 15.400 | 0.0052 |
| *Dendrocopos major* (Linnaeus, 1758) | Buntspecht | Great spotted  woodpecker | Picidae |  |  |  | 78 | 0.1685 | 8.218 | 0.0836 |
| *Dryobates minor* (Linnaeus, 1758) | Kleinspecht |  | Picidae |  |  |  | 1 | 0.0022 | 20.000 | 0.0006 |
| *Dryocopus martius* (Linnaeus, 1758) | Schwarzspecht |  | Picidae |  |  |  | 11 | 0.0238 | 9.636 | 0.0133 |
| *Emberiza citrinella* (Linnaeus, 1758) | Goldammer |  |  |  | x | x | 5 | 0.0108 | 13.200 | 0.0041 |
| *Erithacus rubecula* (Linnaeus, 1758) | Rotkehlchen | Robin |  |  | x |  | 126 | 0.2721 | 6.968 | 0.1526 |
| *Falco peregrinus* (Tunstall, 1771) | Wanderfalke |  | Falco spp. |  | x |  | 6 | 0.0130 | 10.667 | 0.0080 |
| *Falco* spp. | Falken | Falcons |  |  | x |  | 87 | 0.1879 | 8.333 | 0.0971 |
| *Falco tinnunculus* (Linnaeus, 1758) | Turmfalke |  | Falco spp. |  | x |  | 18 | 0.0389 | 11.500 | 0.0199 |
| *Ficedula hypoleuca* (Pallas, 1764) | Trauerschnäpper |  |  |  |  |  | 2 | 0.0043 | 18.500 | 0.0016 |
| *Fringilla coelebs* (Linnaeus, 1758) | Buchfink |  | Fringillidae |  | x |  | 21 | 0.0454 | 10.095 | 0.0217 |
| Fringillidae | Finken | Finches |  |  | x |  | 120 | 0.2592 | 7.875 | 0.1358 |
| *Fulica atra* (Linnaeus, 1758) | Blässhuhn |  |  |  |  |  | 6 | 0.0130 | 17.667 | 0.0028 |
| *Gallinula chloropus* (Linnaeus, 1758) | Teichralle |  |  |  |  |  | 2 | 0.0043 | 8.500 | 0.0017 |
| *Garrulus glandarius* (Linnaeus, 1758) | Eichelhäher | Jay |  |  | x |  | 56 | 0.1210 | 7.196 | 0.0722 |
| *Grus grus* (Linnaeus, 1758) | Kranich | Crane |  |  | x |  | 87 | 0.1879 | 10.506 | 0.0740 |
| *Haematopus ostralegus* (Linnaeus, 1758) | Austernfischer |  |  |  | x |  | 2 | 0.0043 | 17.000 | 0.0017 |
| *Haliaeetus albicilla* (Linnaeus, 1758) | Seeadler |  | Aquila chrysaetos/ Pandion haliaetus/ Haliaeetus albicilla |  | x |  | 11 | 0.0238 | 13.000 | 0.0088 |
| Hirundinidae | Schwalben | Swallows |  |  | x |  | 89 | 0.1922 | 7.180 | 0.1058 |
| *Hirundo rustica* (Linnaeus, 1758) | Rauchschwalbe |  | Hirundinidae |  | x | x | 6 | 0.0130 | 14.500 | 0.0066 |
| *Lanius collurio* (Linnaeus, 1758) | Neuntöter |  |  |  | x | x | 2 | 0.0043 | 14.500 | 0.0011 |
| Laridae | Möwen | Gulls |  |  | x |  | 50 | 0.1080 | 8.860 | 0.0465 |
| *Larus argentatus* (Pontoppidan, 1763) | Silbermöwe |  | Laridae |  | x |  | 1 | 0.0022 | 9.000 | 0.0016 |
| *Leptoptilos crumeniferus* (Lesson, RP, 1831) | Marabu |  |  |  |  |  | 1 | 0.0022 | 15.000 | 0.0004 |
| *Linaria cannabina* (Linnaeus, 1758) | Bluthänfling |  | Fringillidae |  | x | x | 4 | 0.0086 | 16.750 | 0.0025 |
| *Lullula arborea* (Linnaeus, 1758) | Heidelerche |  |  |  |  |  | 2 | 0.0043 | 21.500 | 0.0010 |
| *Luscinia megarhynchos* (Brehm, 1831) | Nachtigall |  |  |  |  |  | 15 | 0.0324 | 9.200 | 0.0163 |
| *Luscinia svecica* (Linnaeus, 1758) | Blaukehlchen |  |  |  |  |  | 1 | 0.0022 | 3.000 | 0.0015 |
| *Melopsittacus undulatus* (Shaw, 1805) | Wellensittich |  |  |  |  |  | 1 | 0.0022 | 2.000 | 0.0019 |
| *Merops apiaster* (Linnaeus, 1758) | Bienenfresser |  |  |  |  |  | 5 | 0.0108 | 7.400 | 0.0062 |
| *Milvus migrans* (Boddaert, 1783) | Schwarzmilan |  |  | Accipitriformes | x |  | 3 | 0.0065 | 7.333 | 0.0039 |
| *Milvus milvus* (Linnaeus, 1758) | (Rot-)Milan | Red kite |  | Accipitriformes | x | x | 146 | 0.3153 | 7.486 | 0.1664 |
| *Motacilla alba* (Linnaeus, 1758) | Bachstelze |  |  |  | x |  | 18 | 0.0389 | 6.944 | 0.0246 |
| *Motacilla flava* (Linnaeus, 1758) | Schafstelze |  |  |  | x | x | 2 | 0.0043 | 12.000 | 0.0016 |
| *Oriolus oriolus* (Linnaeus, 1758) | Pirol |  |  |  |  |  | 7 | 0.0151 | 6.571 | 0.0113 |
| *Otis tarda* (Linnaeus, 1758) | Großtrappe |  |  |  |  | x | 3 | 0.0065 | 10.333 | 0.0018 |
| *Pandion haliaetus* (Linnaeus, 1758) | Fischadler |  | Aquila chrysaetos/ Pandion haliaetus/ Haliaeetus albicilla | Accipitriformes | x |  | 8 | 0.0173 | 11.750 | 0.0079 |
| *Aquila chrysaetos*/ *Pandion haliaetus*/ *Haliaeetus albicilla* (Linnaeus, 1758) | Adler | Eagle |  |  | x |  | 43 | 0.0929 | 9.093 | 0.0435 |
| Paridae | Meisen | Tits |  |  |  |  | 253 | 0.5464 | 4.494 | 0.3884 |
| *Parus cristatus* (Linnaeus, 1758) | Haubenmeise |  | Paridae |  |  |  | 1 | 0.0022 | 11.000 | 0.0009 |
| *Parus major* (Linnaeus, 1758) | Kohlmeise | Great tit | Paridae |  |  |  | 104 | 0.2246 | 6.673 | 0.1377 |
| *Passer domesticus* (Linnaeus, 1758) | Haussperling |  | Passer spp. |  | x | x | 6 | 0.0130 | 8.833 | 0.0090 |
| *Passer montanus* (Linnaeus, 1758) | Feldsperling |  | Passer spp. |  | x | x | 6 | 0.0130 | 4.667 | 0.0100 |
| *Passer* spp. | Spatzen | Sparrows |  |  | x | x | 338 | 0.7300 | 4.104 | 0.5179 |
| *Pelecanus* spp. | Pelikane |  |  |  |  |  | 1 | 0.0022 | 3.000 | 0.0011 |
| *Perdix perdix* (Linnaeus, 1758) | Rebhuhn |  |  |  | x | x | 21 | 0.0454 | 10.048 | 0.0212 |
| *Phalacrocorax carbo* (Linnaeus, 1758) | Kormoran |  |  |  |  |  | 7 | 0.0151 | 14.000 | 0.0047 |
| *Phasianus colchicus* (Linnaeus, 1758) | Fasan | Pheasant |  |  | x | x | 35 | 0.0756 | 12.457 | 0.0250 |
| *Phoenicopterus roseus* (Pallas, 1811) | Flamingo |  |  |  |  |  | 1 | 0.0022 | 3.000 | 0.0016 |
| *Phoenicurus ochruros* (Gmelin, S.G., 1774) | Hausrotschwanz |  | Phoenicurus spp. |  |  |  | 4 | 0.0086 | 8.750 | 0.0063 |
| *Phoenicurus phoenicurus* (Linnaeus, 1758) | Gartenrotschwanz |  | Phoenicurus spp. |  | x | x | 5 | 0.0108 | 10.000 | 0.0068 |
| *Phoenicurus* spp. | Rotschwänzchen | Redstart |  |  | x |  | 52 | 0.1123 | 6.231 | 0.0733 |
| *Phylloscopus collybita* (Vieillot, 1817) | Zilpzalp |  |  |  |  |  | 2 | 0.0043 | 8.500 | 0.0011 |
| *Pica pica* (Linnaeus, 1758) | Elster | Magpie |  |  | x |  | 143 | 0.3089 | 6.811 | 0.1716 |
| Picidae | Spechte | Woodpeckers |  |  |  |  | 143 | 0.3089 | 6.483 | 0.1802 |
| *Picus viridis* (Linnaeus, 1758) | Grünspecht | Green woodpecker | Picidae |  |  |  | 28 | 0.0605 | 9.500 | 0.0298 |
| *Podiceps cristatus* (Linnaeus, 1758) | Haubentaucher |  |  |  |  |  | 7 | 0.0151 | 14.429 | 0.0050 |
| *Poecile montanus* (Conrad von Baldenstein, 1827) | Weidenmeise |  | Paridae |  |  |  | 1 | 0.0022 | 8.000 | 0.0012 |
| *Poecile palustris* (Linnaeus, 1758) | Sumpfmeise |  | Paridae |  |  |  | 1 | 0.0022 | 5.000 | 0.0019 |
| *Pyrrhula pyrrhula* (Linnaeus, 1758) | Gimpel/Dompfaff |  | Fringillidae |  | x |  | 4 | 0.0086 | 15.000 | 0.0025 |
| Rallidae | Rallen |  |  |  | x |  | 1 | 0.0022 | 3.000 | 0.0013 |
| *Regulus regulus* (Linnaeus, 1758) | Wintergoldhähnchen |  | Regulus spp. |  |  |  | 1 | 0.0022 | 14.000 | 0.0006 |
| *Regulus* spp. | Goldhähnchen |  |  |  |  |  | 2 | 0.0043 | 13.000 | 0.0020 |
| *Riparia riparia* (Linnaeus, 1758) | Uferschwalbe |  | Hirundinidae |  | x |  | 1 | 0.0022 | 10.000 | 0.0008 |
| *Saxicola rubetra* (Linnaeus, 1758) | Braunkehlchen |  |  |  | x | x | 1 | 0.0022 | 3.000 | 0.0020 |
| *Sitta europaea* (Linnaeus, 1758) | Kleiber |  |  |  |  |  | 16 | 0.0346 | 9.813 | 0.0188 |
| *Spinus spinus* (Linnaeus, 1758) | Zeisig |  | Fringillidae |  |  |  | 5 | 0.0108 | 12.000 | 0.0039 |
| *Streptopelia decaocto* (Frivaldszky, 1838) | Türkentaube |  | Columbidae |  |  |  | 5 | 0.0108 | 15.600 | 0.0027 |
| *Streptopelia turtur* (Linnaeus, 1758) | Turteltaube |  | Columbidae |  | x |  | 1 | 0.0022 | 21.000 | 0.0009 |
| Strigidae | Kauze |  |  |  | x |  | 13 | 0.0281 | 11.154 | 0.0109 |
| Strigiformes | Eulen | Owls |  |  | x |  | 55 | 0.1188 | 9.255 | 0.0511 |
| *Strix aluco* (Linnaeus, 1758) | Waldkauz |  | Strigidae |  |  |  | 4 | 0.0086 | 8.750 | 0.0034 |
| *Sturnus vulgaris* (Linnaeus, 1758) | Star | Starling |  |  | x | x | 126 | 0.2721 | 6.183 | 0.1545 |
| *Sylvia atricapilla* (Linnaeus, 1758) | Mönchsgrasmücke |  | Sylvia spp. |  | x |  | 3 | 0.0065 | 13.333 | 0.0027 |
| *Sylvia* spp. | Grasmücken |  |  |  | x |  | 4 | 0.0086 | 10.500 | 0.0045 |
| *Tetrao urogallus* (Linnaeus, 1758) | Auerhahn |  |  |  |  |  | 4 | 0.0086 | 7.250 | 0.0045 |
| Trochilidae | Kolibri |  |  |  |  |  | 1 | 0.0022 | 17.000 | 0.0001 |
| *Troglodytes troglodytes* (Linnaeus, 1758) | Zaunkönig |  |  |  | x |  | 17 | 0.0367 | 12.000 | 0.0180 |
| *Turdus merula* (Linnaeus, 1758) | Amsel | Blackbird |  |  |  |  | 243 | 0.5248 | 4.654 | 0.3656 |
| *Turdus philomelos* (Brehm, 1831) | Singdrossel |  | Turdus spp. |  | x | x | 5 | 0.0108 | 13.600 | 0.0051 |
| *Turdus pilaris* (Linnaeus, 1758) | Wacholderdrossel |  | Turdus spp. |  | x | x | 1 | 0.0022 | 13.000 | 0.0008 |
| *Turdus* spp. | Drosseln | Thrushes |  |  | x | x | 66 | 0.1425 | 6.818 | 0.0850 |
| *Tyto alba* (Scopoli, 1769) | Schleiereule |  | Strigiformes |  | x |  | 2 | 0.0043 | 20.500 | 0.0012 |
| *Upupa epops* (Linnaeus, 1758) | Wiedehopf |  |  |  | x | x | 10 | 0.0216 | 10.900 | 0.0098 |
| *Vanellus Vanellus* (Linnaeus, 1758) | Kiebitz |  |  |  | x | x | 13 | 0.0281 | 12.000 | 0.0122 |
| - | Teichmeise |  |  |  |  |  | 1 | 0.0022 | 9.000 | 0.0016 |

^1^ Scientific bird names according to Avilist – The Global Avian Checklist (<https://www.avilist.org/>)

^2^ Name used by most of the participants

^3^ Only for those taxa in the cultural domain, i.e. with a relative frequency ≥ 0.05.
