# Supplementary figures and images for: Young adults in eastern Germany know dandelion and sparrows but few farmland species
Source: J Ethnobiol Ethnomed. 2026 May 14;22:51. doi: 10.1186/s13002-026-00908-2 (PMC13185424; doi:10.1186/s13002-026-00908-2)

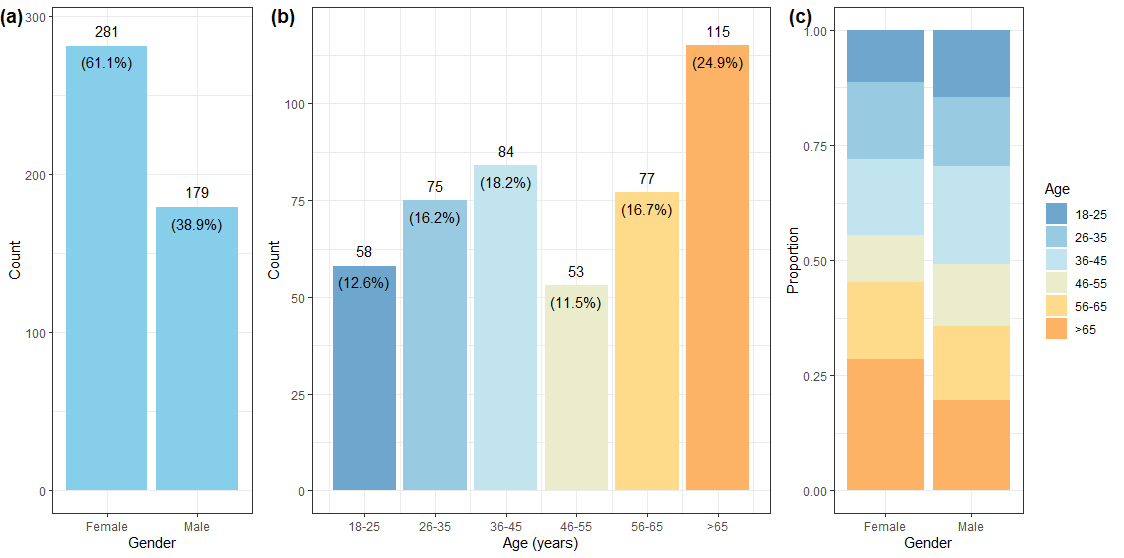


Additional file 4. Distribution of gender and age in the sample of 463 participants.

Supplement: Supplementary file 4 — Supplementary Material 4 [file 13002_2026_908_MOESM4_ESM.docx]

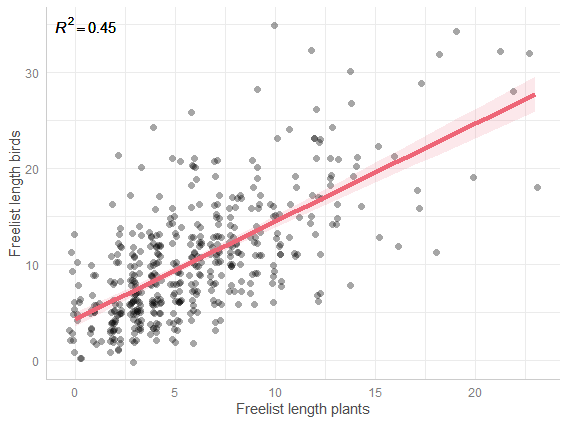


Additional file 5. Correlation between freelist lengths for plants and birds.

Supplement: Supplementary file 5 — Supplementary Material 5 [file 13002_2026_908_MOESM5_ESM.docx]
