# Supplementary material for: Young adults in eastern Germany know dandelion and sparrows but few farmland species
Source: J Ethnobiol Ethnomed. 2026 May 14;22:51. doi: 10.1186/s13002-026-00908-2 (PMC13185424; doi:10.1186/s13002-026-00908-2)
Supplement: Supplementary file 6 — Supplementary Material 6 [file 13002_2026_908_MOESM6_ESM.docx]

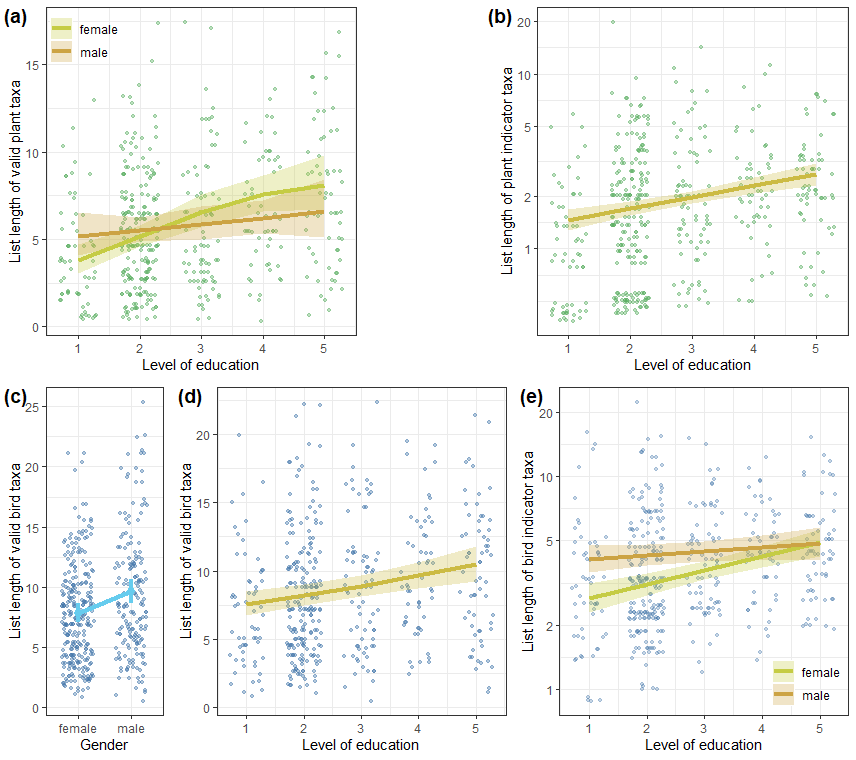


Additional file 6. Partial effects of gender and the level of education on the freelist length for valid plant taxa (a), plant indicator taxa (b), valid bird taxa (c, d), and bird indicator taxa (e) according to generalized additive models (Table 1 in the main text). Shown are partial residuals, the regression line and the 95% confidence band.
